# Increased core body temperature exacerbates defective protein prenylation in mouse models of mevalonate kinase deficiency

**DOI:** 10.1172/JCI160929

**Published:** 2022-10-03

**Authors:** Marcia A. Munoz, Oliver P. Skinner, Etienne Masle-Farquhar, Julie Jurczyluk, Ya Xiao, Emma K. Fletcher, Esther Kristianto, Mark P. Hodson, Seán I. O’Donoghue, Sandeep Kaur, Robert Brink, David G. Zahra, Elissa K. Deenick, Kristen A. Perry, Avril A.B. Robertson, Sam Mehr, Pravin Hissaria, Catharina M. Mulders-Manders, Anna Simon, Michael J. Rogers

**Affiliations:** 1 Garvan Institute of Medical Research and School of Clinical Medicine, UNSW Sydney, Sydney, New South Wales, Australia.; 2 Victor Chang Cardiac Innovation Centre, Victor Chang Cardiac Research Institute, Sydney, New South Wales, Australia.; 3 School of Pharmacy, University of Queensland, Woolloongabba, Queensland, Australia.; 4 School of Chemistry and Molecular Biosciences, University of Queensland, Brisbane, Queensland, Australia.; 5 Royal Children’s Hospital, Melbourne, Victoria, Australia.; 6 Royal Adelaide Hospital, SA Pathology and University of Adelaide, Adelaide, South Australia, Australia.; 7 Department of Internal Medicine, Radboudumc Expertise Centre for Immunodeficiency and Autoinflammation, Radboud University Medical Centre, Nijmegen, Netherlands.

**Keywords:** Inflammation, Metabolism, Autoimmune diseases, Macrophages, Monogenic diseases

## Abstract

Mevalonate kinase deficiency (MKD) is characterized by recurrent fevers and flares of systemic inflammation, caused by biallelic loss-of-function mutations in *MVK*. The underlying disease mechanisms and triggers of inflammatory flares are poorly understood because of the lack of in vivo models. We describe genetically modified mice bearing the hypomorphic mutation p.Val377Ile (the commonest variant in patients with MKD) and amorphic, frameshift mutations in *Mvk*. Compound heterozygous mice recapitulated the characteristic biochemical phenotype of MKD, with increased plasma mevalonic acid and clear buildup of unprenylated GTPases in PBMCs, splenocytes, and bone marrow. The inflammatory response to LPS was enhanced in compound heterozygous mice and treatment with the NLRP3 inflammasome inhibitor MCC950 prevented the elevation of circulating IL-1β, thus identifying a potential inflammasome target for future therapeutic approaches. Furthermore, lines of mice with a range of deficiencies in mevalonate kinase and abnormal prenylation mirrored the genotype-phenotype relationship in human MKD. Importantly, these mice allowed the determination of a threshold level of residual enzyme activity, below which protein prenylation is impaired. Elevated temperature dramatically but reversibly exacerbated the deficit in the mevalonate pathway and the defective prenylation in vitro and in vivo, highlighting increased body temperature as a likely trigger of inflammatory flares.

## Graphical abstract

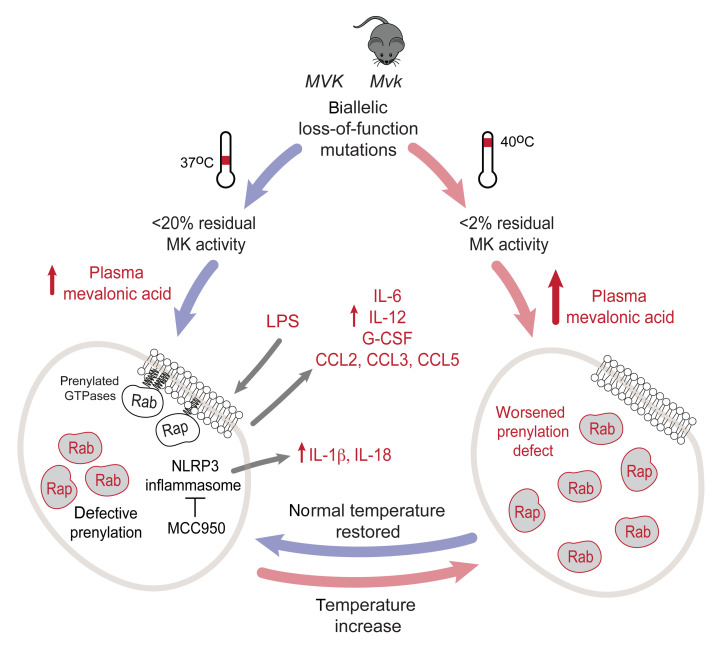

## Introduction

The mevalonate pathway is an essential metabolic pathway required for the biosynthesis of the long-chain isoprenoid lipids farnesyl diphosphate and geranylgeranyl diphosphate (GGPP). The latter are necessary for the posttranslational prenylation of more than 300 proteins, particularly small GTPases such as those of the Rho/Rac/Rap and Rab superfamily (1, 2). Dysregulation of the mevalonate pathway has been associated with a variety of human diseases, and particularly with inflammation. The most striking example is the genetic autoinflammatory disorder mevalonate kinase (MK) deficiency (MKD). MKD is an inborn error of metabolism caused by autosomal recessive inheritance of mutations in *MVK* (3, 4). This gene encodes MK [ATP:(*R*)-mevalonate 5-phosphotransferase, EC 2.7.1.36], a proximal enzyme in the mevalonate pathway (5, 6).

MKD encompasses a severe form of the disease, mevalonic aciduria (OMIM 610377), and a milder periodic fever syndrome, hyperimmunoglobulinemia D syndrome (HIDS, OMIM 260920) (7, 8). In HIDS, the inflammatory symptoms appear in early childhood and feature regular episodes of high fever and systemic inflammation separated by intervals of normal health. The most frequent pathogenic variant in HIDS is a G>A missense mutation resulting in the amino acid substitution p.Val377Ile (5, 7, 9, 10). Individuals homozygous or compound heterozygous for p.V377I have 1% to 20% residual MK enzyme activity (11, 12), but why such a conservative amino acid substitution affects the MK enzyme remains unknown. Mevalonic aciduria is caused by homozygous or compound heterozygous *MVK* variants that affect enzyme function or expression more severely than p.V377I (6), and is associated with extremely low (<0.5%) or undetectable residual MK activity (13–15). In contrast to HIDS, mevalonic aciduria patients present with persistent systemic inflammation (7, 9) as well as neurologic impairment (including cerebellar atrophy, dystonia, and ataxia). The buildup of the MK substrate, mevalonic acid (MA, or the lactone derivative), in urine and plasma is a characteristic feature of mevalonic aciduria and also occurs during inflammatory flares in HIDS (12–16).

Other hallmarks of MKD are elevated serum levels of IL-1β and increased release of IL‑1β from peripheral blood mononuclear cells (PBMCs) and fibroblasts of patients (17, 18), likely due to increased inflammasome activation. We also recently showed that prenylation of Rab and Rap1A GTPases is deficient in MKD PBMCs and that accumulation of unprenylated Rab proteins (uRabs) could be a diagnostic indicator of MKD (19, 20). How loss of MK activity and disruption of the mevalonate pathway lead to enhanced inflammasome activity remains unclear but appears to result from loss of synthesis of GGPP (21, 22) necessary for protein prenylation. Furthermore, the identity of the inflammasome(s) affected by defective prenylation is still controversial, with evidence for enhanced activation of the NLRP3 inflammasome (23) as well as the pyrin inflammasome (24, 25). Also, inflammatory flares in MKD are commonly triggered by stress, strenuous exercise, vaccinations, or infection (5, 12, 26, 27), but the underlying mechanisms remain unknown. Mutations in *MVK* may lead to temperature-sensitive changes in folding or stability of the enzyme (8, 28). Hence, elevations in body temperature could potentially further compromise MK activity and exacerbate the underlying defect in protein prenylation, but this remains to be tested in vivo in a relevant physiological model.

A major reason for the poor understanding of the mechanisms of disease in MKD is the lack of genetic animal models, since complete loss of *Mvk* expression is lethal in homozygous *Mvk*-knockout mice (29). One study used conditional knockout of a prenyltransferase gene to prevent protein prenylation (24). Other approaches utilized pharmacologic inhibitors such as statins, bisphosphonates, and prenyltransferase inhibitors. These compounds, although blocking different steps of the mevalonate pathway, act commonly to inhibit downstream protein prenylation (30), and thus have been used as models of MKD (21–23, 31–36). However, whether any of these pharmacological or genetic models accurately mimic the defect in prenylation that occurs in MKD is not known. An important limitation of these models is that they do not permit studies on complex physiological mechanisms that may affect the mutant MK enzyme and thus contribute to inflammatory flares. To fill this fundamental knowledge gap and better understand the link between defective protein prenylation and inflammatory flares, we used CRISPR/Cas9 gene editing to create physiologically relevant mouse models of MKD carrying biallelic mutations in *Mvk*, including the commonest pathogenic variant V377I.

## Results

### Mvk-mutant mice have reduced MK activity.

Using CRISPR/Cas9 gene editing of C57BL/6J mouse embryos, we created mice with a G>A missense mutation resulting in the substitution of valine in position 377 with isoleucine (p.V377I), and 3 mouse lines carrying heterozygous deletions of 8 bp (Δ*8*), 13 bp (Δ*13*), or 91 bp (Δ*91*) in exon 11 (Supplemental Figure 1; supplemental material available online with this article; https://doi.org/10.1172/JCI160929DS1). All deletions constitute frameshift mutations: Δ*8*, following the codon for T370, results in a predicted extension of 24 amino acids at the C‑terminus; Δ*13*, after the codon for A734, causes premature termination of the C‑terminus; and Δ*91*, the loss of residues L348–S378, alters the sequence of 7 amino acids (residues 348–354, LEQPEVE > PHTQLQL) and causes deletion of all remaining residues from 355 onwards (Supplemental Figure 1).

**Figure 1:**
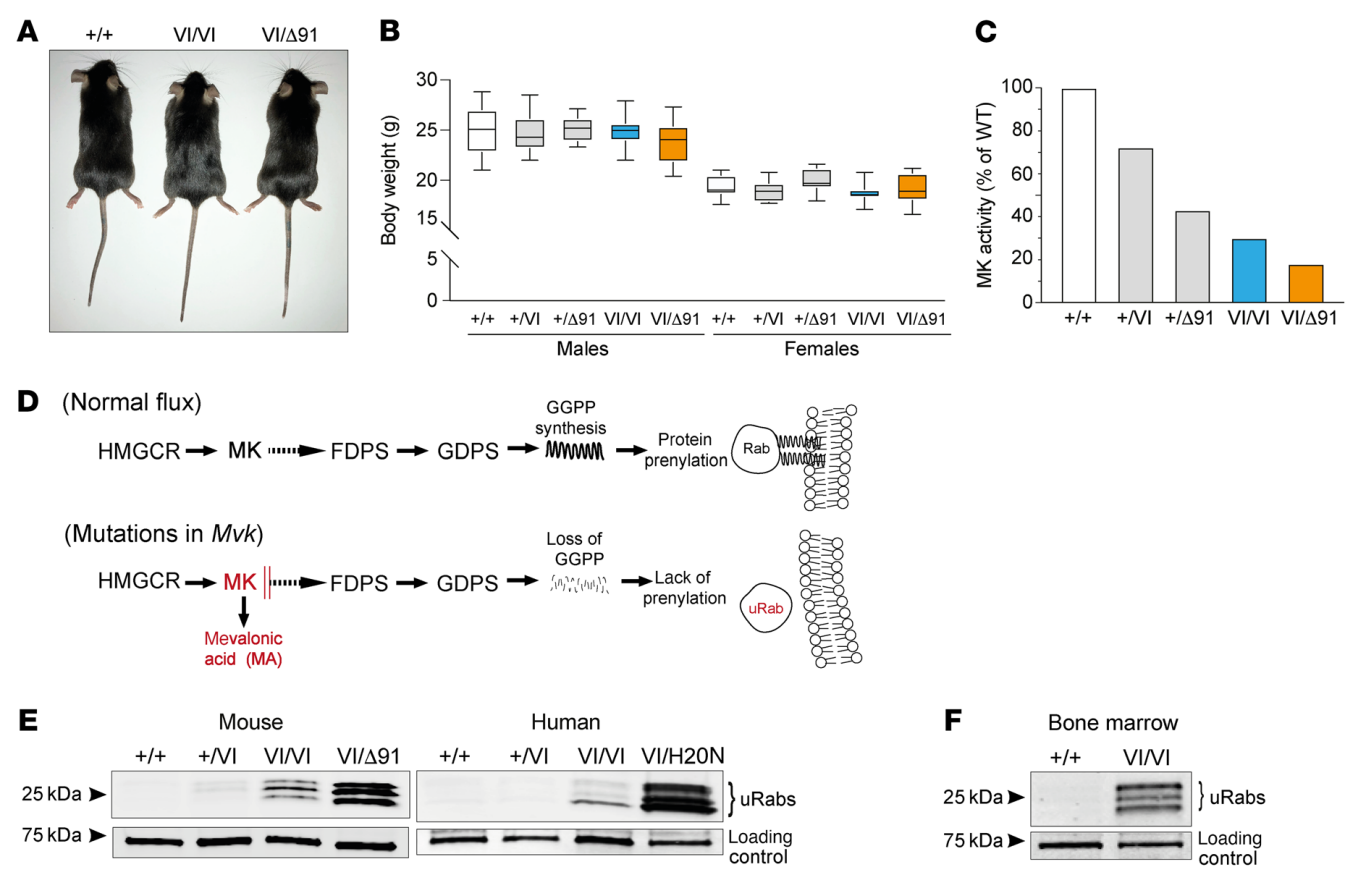
*Mvk^VI/VI^* and *Mvk*^VI/Δ91^ mice recapitulate the defective prenylation phenotype of mevalonate kinase deficiency in humans. (**A**) Appearance of 12-week-old wild-type and *Mvk*-mutant mice. (**B**) Body weights of male (*n =* 12) and female (*n =* 11) mice (box shows median and first and third quartiles, whiskers show min and max). (**C**) Mevalonate kinase (MK) activity in liver homogenates, expressed as a percentage of activity in wild-type (values representative of measurements in 2 mice per genotype). (**D**) Diagram of the mevalonate pathway normally leading to protein prenylation; lack of MK activity leads to buildup of mevalonic acid, loss of geranylgeranyl diphosphate (GGPP) synthesis, and accumulation of unprenylated Rab (uRab) proteins. HMGCR, HMG-CoA reductase; FDPS, farnesyl diphosphate synthase; GDPS, geranylgeranyl diphosphate synthase. A biochemical in vitro assay based on the detection of uRabs was used to measure the defect in protein prenylation. (**E**) Comparison of uRabs in spleen cells from *Mvk^VI/VI^* (VI/VI) and *Mvk*^VI/Δ91^ (VI/Δ91) mice and in PBMCs from MKD patients (genotypes *MVK^V377I/V377I^* and *MVK^V377I/H20N^*). Wild-type and heterozygous genotypes were used as controls, and an endogenous, biotinylated 73 kDa protein as loading control. (**F**) Detection of uRabs in bone marrow from homozygous *Mvk^VI/VI^* (VI/VI) mice.

*Mvk*-mutant mice were intercrossed to create animals homozygous for *Mvk^V377I^* (hereafter abbreviated *Mvk^VI/VI^* or simply VI/VI). Compound heterozygous mice were generated with the biallelic mutations *Mvk^VI^* and either Δ*8*, Δ*13*, or Δ*91* (i.e., *Mvk*^VI/Δ8^, *Mvk*^VI/Δ13^, or *Mvk*^VI/Δ91^, respectively). *Mvk^VI/VI^*, *Mvk*^VI/Δ8^, *Mvk*^VI/Δ13^, and *Mvk*^VI/Δ91^ mice were viable, born in the expected Mendelian ratios, and did not differ in appearance from wild-type or littermate heterozygous mice (Figure 1, A and B). No homozygous offspring with Δ*8*, Δ*13*, or Δ*91* deletions were born, indicating that these were amorphic mutations. MK is highly expressed in liver. However, no C-terminal-truncated form of MK of the predicted mass could be detected in liver from Δ*91* mice by Western blotting with an antibody that binds to the mid-region of MK (Supplemental Figure 2), suggesting lack of expression or increased degradation of the mutant Δ*91* MK protein.

Consistent with the Δ*91* mutation causing complete loss of function, heterozygous *Mvk*^+/Δ91^ mice had approximately 50% normal MK activity in liver homogenates compared with wild-type counterparts (Figure 1C). Furthermore, animals carrying the milder p.V377I mutation in 1 allele (*Mvk^+/VI^*) had 73% residual MK activity, whereas *Mvk^VI/VI^* homozygous mice had 19%. Importantly, *Mvk*^VI/Δ91^ mice had the lowest MK enzyme activity (9%) (Figure 1C). Similar results were obtained with bone marrow (BM) extracts, with 18% and 5% residual MK activity in *Mvk^VI/VI^* and *Mvk*^VI/Δ91^ BM, respectively. Together, these results suggest that a single *Mvk^VI^* allele contributes approximately 10% residual MK activity in vivo.

### Mutations in MK cause a similar pattern of defective protein prenylation in immune cells in mice and humans.

In humans, mutations in *MVK* that reduce MK activity lead to decreased synthesis of isoprenoid lipids via the mevalonate pathway (Figure 1D) and hence deficient protein prenylation (19, 20, 36). We analyzed immune cells from *Mvk*-mutant mice for evidence of defective protein prenylation, using an in vitro biochemical assay (30, 36, 37) to measure the buildup of uRabs. Unprenylated small GTPases are almost undetectable under normal conditions. Accordingly, wild-type and heterozygous *Mvk^+/VI^*, *Mvk*^+/Δ8^, *Mvk*^+/Δ13^, and *Mvk*^+/Δ91^ animals showed no accumulation of uRabs in spleen and BM cells (Figure 1E and Supplemental Figure 3, A–C), while in homozygous *Mvk^VI/VI^* cells there was a cluster of bands of 23–27 kDa corresponding to uRabs (Figure 1E and Supplemental Figure 3A). This mild prenylation defect was more obvious when the sensitivity of the assay was enhanced by increasing the incubation time with Rab GTPase (Figure 1F). By contrast, cells from compound heterozygous *Mvk*^VI/Δ91^ mice had a robust buildup of uRabs (spleen, Figure 1E; BM and PBMCs, Supplemental Figure 3, A and D) to levels almost 20 times higher than in *Mvk^VI/VI^* cells (Supplemental Figure 3B). *Mvk*^VI/Δ91^ BM cells also showed a clear accumulation of unprenylated Rap1A (uRap1A) detectable by Western blotting (Supplemental Figure 3A). Similar levels of uRabs and uRap1A were found in cells from compound heterozygous *Mvk*^VI/Δ8^ and *Mvk*^VI/Δ13^ animals (Supplemental Figure 3, A and B). We therefore chose to focus on *Mvk*^VI/Δ91^ mice as representative of the compound heterozygous phenotype in further studies.

To compare the extent of the prenylation defect in *Mvk*-mutant mice and MKD patients, we used freshly isolated PBMCs from individuals homozygous for p.V377I or compound heterozygous for p.V377I and p.H20N variants. Importantly, the p.H20N mutation is in a highly conserved hotspot region (residues 8–35, around the active site of MK) in which mutations are predicted to severely affect enzyme activity (38). We found that the pattern of defective prenylation in spleen cells from *Mvk*-mutant mice bore striking similarity to that in patient PBMCs. In other words, mice and humans with comparable genotypes had similar prenylation defects: a mild defect (homozygous p.V377I) or more pronounced (compound heterozygous with biallelic combination of p.V377I with a more severe mutation as in murine *Mvk*^VI/Δ91^ or human *MVK^V377I/H20N^*) (Figure1E).

### Mvk^VI/Δ91^ mice have elevated MA in plasma and cell extracts.

**Figure 2:**
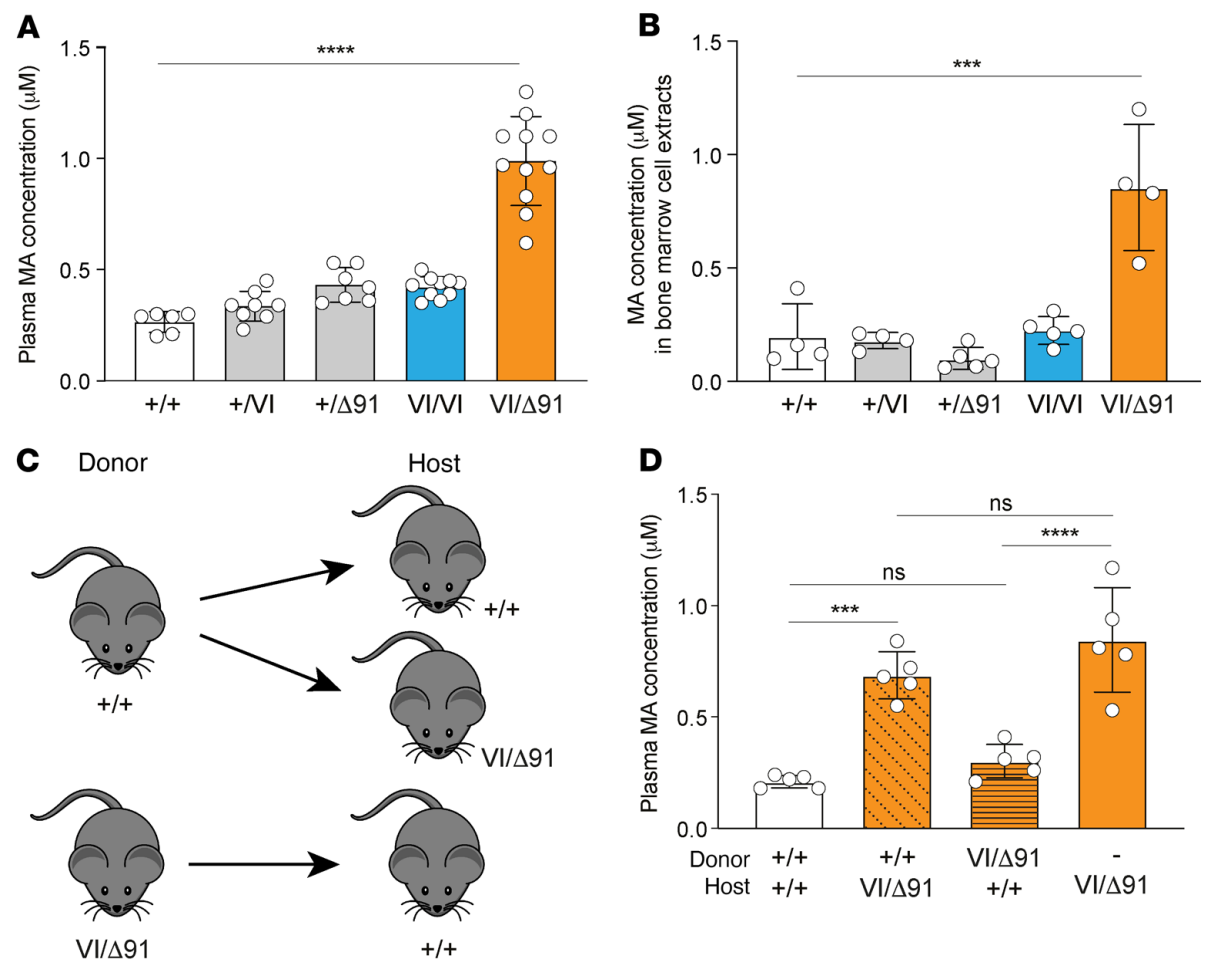
*Mvk*^VI/Δ91^ mice have elevated plasma mevalonic acid from nonhematopoietic tissue. (**A**) Concentration of mevalonic acid (MA) in plasma, or (**B**) in bone marrow extracts, from wild-type and *Mvk*-mutant mice. (**C**) Scheme of bone marrow transfer from donor mice (wild-type or *Mvk*^VI/Δ91^) to generate chimeric host mice. (**D**) Concentration of MA in plasma from chimeric mice and *Mvk*^VI/Δ91^ control mice. Bars show mean ± SD (*n* = 6–11 mice per genotype in **A**, *n =* 4–5 mice per group in **B** and **D**; each symbol represents a single mouse). ***P < 0.001; ****P < 0.0001 by 1-way ANOVA with Tukey’s post hoc test. Data in **D** are representative of 2 independent experiments.

Lack of MK activity causes the buildup of the substrate MA (Figure 1D). Liquid chromatography–tandem mass spectrometry (LC-MS/MS) analysis of plasma revealed significantly higher levels of MA in *Mvk*^VI/Δ91^ compound heterozygous mice compared with wild-type *Mvk^+/+^* animals. Plasma MA was similar between heterozygous *Mvk^+/VI^*, *Mvk*^+/Δ91^, and homozygous *Mvk^VI/VI^* mice (Figure 2A). Likewise, levels of intracellular MA were not different in BM cell extracts from *Mvk^+/+^*, *Mvk^+/VI^*, *Mvk*^+/Δ91^, or *Mvk^VI/VI^* animals but significantly higher in cell extracts from *Mvk*^VI/Δ91^ mice (Figure 2B).

We used chimeric mice (Figure 2C) to determine the contribution of the hematopoietic BM cell compartment to the high levels of MA in plasma from *Mvk*^VI/Δ91^ animals. Plasma MA levels remained low in wild-type hosts receiving *Mvk*^VI/Δ91^ mutant BM, and comparable to nonchimeric wild-type controls 8 weeks after BM transfer (Figure 2D). In contrast, *Mvk*^VI/Δ91^ recipients of wild-type BM retained significantly elevated plasma MA, similar to nonchimeric *Mvk*^VI/Δ9^ animals (Figure 2D). Hence, while BM cells may contribute a small amount to total plasma MA, nonhematopoietic tissues appear to be the major source of plasma MA in *Mvk*^VI/Δ91^ mice.

### p.V377I and Δ91 mutations affect a highly conserved core region of the MK protein.

We used Aquaria, a publicly available molecular graphics resource (39, 40), to gain insights into how the *Mvk^VI^* and *Mvk*^Δ91^ mutations affect MK enzyme activity. MK had a total of 119 related 3D structures. The closest matches were 2 rat MK structures (88% homology to mouse MK and an HHblits *E* value of 3 × 10^–46^) (41). For further comparisons, we chose Protein Data Bank (PDB) entry 1kvk-A (42), since it also contains a bound ATP substrate.

**Figure 3:**
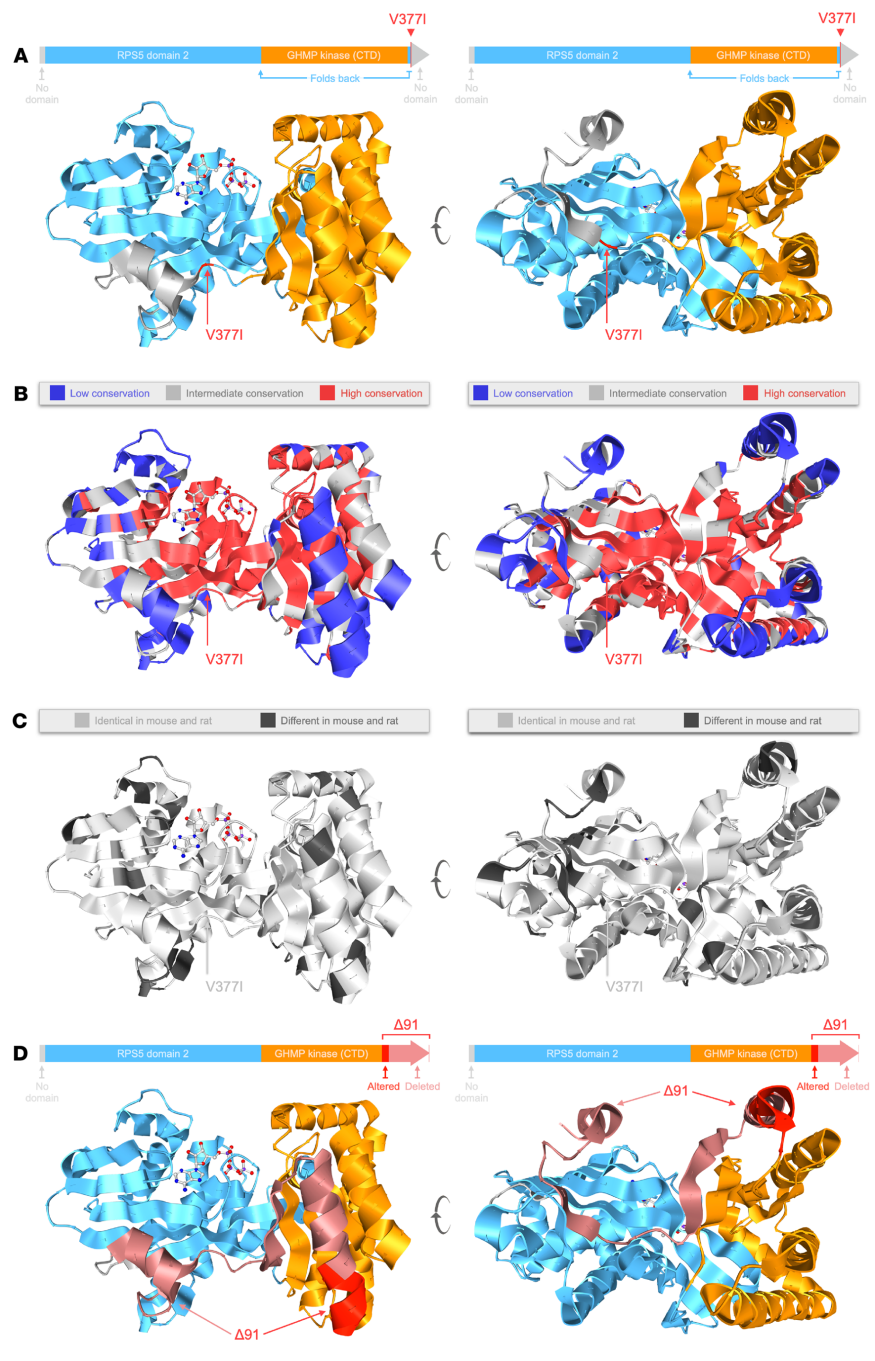
Visualization of the conserved core region of mevalonate kinase affected by p.V377I and Δ*91* mutations. The figures show the crystal structure of a monomer of rat mevalonate kinase from PDB entry 1kvk-A, visualized using Aquaria. Left and right views are related by approximately 90° rotation on the *y* axis. (**A**) CATH domain assignments (arrows above) show the N-terminal RPS5 domain (blue), GHMP kinase domain (orange), and the position of the Val377 mutation within a 3-residue segment that forms part of the RPS5 domain but is located near the C-terminus. (**B**) Regions of sequence conservation mapped in Aquaria using ConSurf conservation scores: low (blue), intermediate (gray), and high (red) conservation. (**C**) Mouse-to-rat alignments show that almost all residues in the conserved hydrophobic core (including the short segment around Val377) are identical (light gray) in mouse and rat. (**D**) The Δ*91* mutation in *Mvk* alters residues 348–354 and causes premature truncation (deletion of all residues from 355 to the C-terminus, pink).

Aquaria and the CATH (class, architecture, topology, and homologous superfamily) structure classification database were used to identify relevant, evolutionarily conserved regions in MK (43). We found 2 clear domains: (a) a C‑terminal GHMP kinase domain (a family of kinases named after 4 members: galactokinase, homoserine kinase, mevalonate kinase, and phosphomevalonate kinase; CATH superfamily 3.30.70.890) within residues 227–374; and (b) an N‑terminal region spanning residues 7–226 homologous to a ribosomal protein S5 domain 2-type fold (also known as RPS5 domain 2; CATH superfamily 3.30.230.10) with an unusual 3-residue segment close to the C-terminus (375–377). Importantly, Val377 is part of this 3-residue region that folds back to form part of the N‑terminal RPS5 domain (Figure 3A): structure available at https://aquaria.app/Mouse/Mvk/1kvk/A?zenodo.3632187.V377I Thus, the *Mvk^VI^* (p.V377I) mutation would be expected to disrupt functions associated with this domain. Furthermore, the Val377 residue is buried from the solvent and forms part of the highly conserved, hydrophobic core of MK (Figure 3B): structure available at https://aquaria.app/Mouse/Mvk/1kvk/A?zenodo.3632187.conservation Any variants occurring in these core residues, even conservative substitutions as in p.V377I, are expected to be detrimental, and this is consistent with the partial loss of MK activity associated with the *Mvk^VI^* allele. The remaining small segments on the N‑terminus (residues 1–6) and C-terminus (residues 378–395) had no identifiable CATH domain.

The amino acid sequences in the conserved hydrophobic core of MK are almost identical between mouse and rat (Figure 3C): structure available at https://aquaria.app/Mouse/Mvk/1kvk/A?zenodo.3632187.mouse_vs_rat, and, with the exception of a single residue inserted at the C-terminus (Leu396), align with human MK without any gaps. The *Mvk*^Δ91^ mutation is predicted to alter residues 348–354 (LEQPEVE > PHTQLQL) and cause deletion of all remaining amino acids from 355 onwards. These changes involve the highly conserved core as well as both GHMP and RPS5 domains (Figure 3D): structure available at https://aquaria.app/Mouse/Mvk/1kvk/A?zenodo.3632187.Δ91 Thus, the Δ*91* mutation would be expected to severely affect MK activity, and this is consistent with the complete loss of function associated with the *Mvk*^Δ91^ allele, as described above.

### The effect of genetic disruption of Mvk differs from pharmacologic inhibition of the mevalonate pathway.

**Figure 4:**
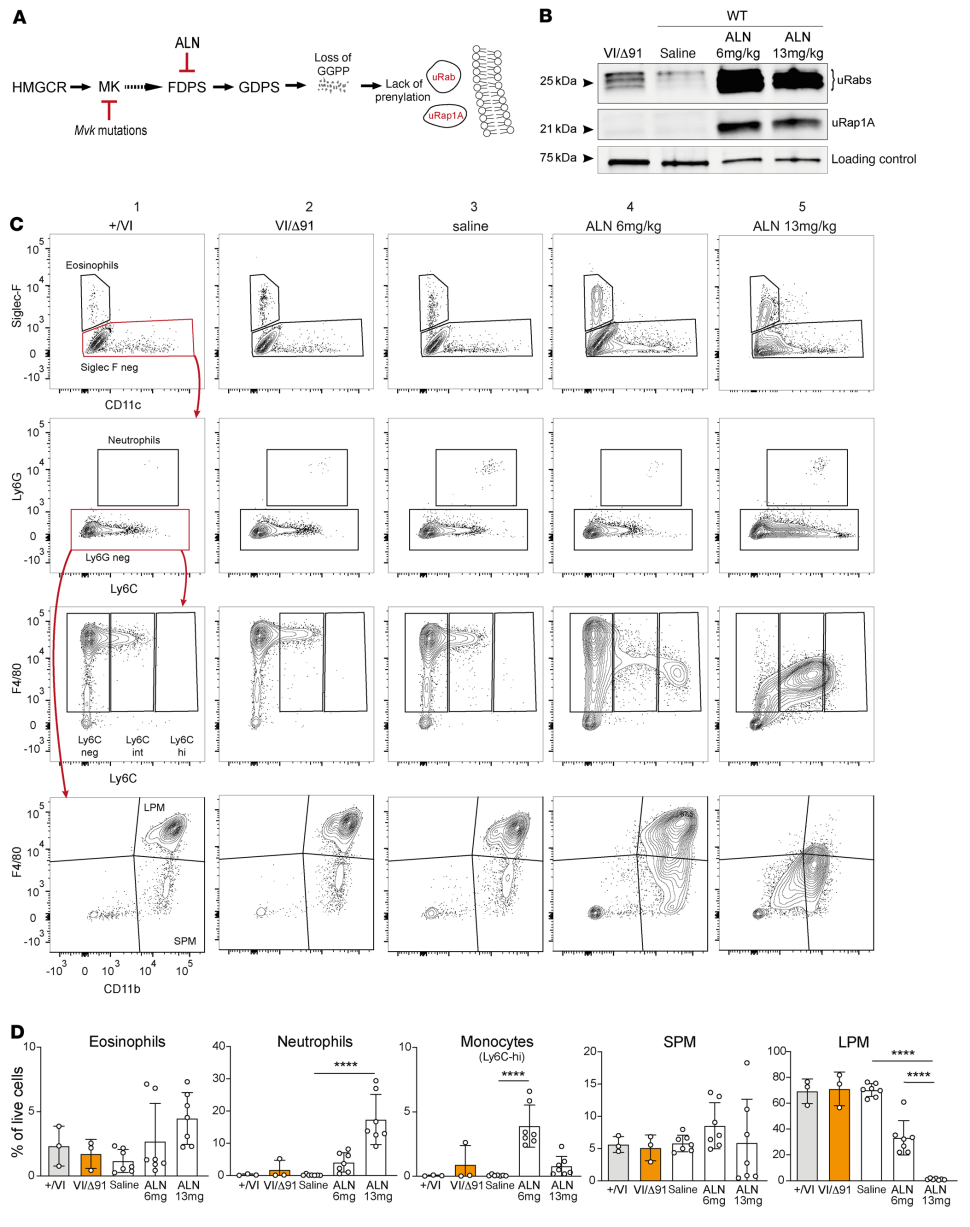
*Mvk*^VI/Δ91^ mice exhibit a lesser defect in prenylation and lack peritoneal inflammation compared with pharmacologic inhibition of the mevalonate pathway. (**A**) Diagram of the mevalonate pathway and points of inhibition in *Mvk*-mutant mice and in mice treated with alendronate (ALN). (**B**) Analysis of unprenylated Rab GTPases (uRabs) and unprenylated Rap1A (uRap1A) in peritoneal cells from *Mvk*^VI/Δ91^ mice, and from wild-type mice 48 hours after i.p. treatment with ALN (6 mg/kg or 13 mg/kg) or saline control. (**C**) Representative FACS plots of peritoneal cells. Rows show gating of eosinophils, neutrophils, monocytes, and large (LPM) and small (SPM) peritoneal macrophages. Columns 1 and 2 show FACS plots from *Mvk^+/VI^* and *Mvk*^VI/Δ91^ mice, columns 3–5 show FACS plots from wild-type mice treated with saline or 6 mg/kg or 13 mg/kg ALN. Polygons in red depict the population displayed in the proceeding plot (red arrows). (**D**) Histograms show relative abundance (percentage of live cell singlets) of eosinophils (TCRβ^–^B220^–^CD11c^–^Siglec-F^+^), neutrophils (TCRβ^–^B220^–^Siglec-F^–^Ly6G^hi^), monocytes (TCRβ^–^B220^–^Siglec-F^–^Ly6G^–^F4/80^+^Ly6C^hi^), LPM (TCRβ^–^B220^–^Siglec-F^–^Ly6G^–^F4/80^hi^CD11b^hi^), and SPM (TCRβ^–^B220^–^Siglec-F^–^Ly6G^–^F4/80^+^CD11b^+^). Bars are the mean ± SD, and each symbol represents a single mouse; *n =* 3 per group for *Mvk^+/VI^* and *Mvk*^VI/Δ91^mice, *n =* 7 per group for ALN- and saline-treated mice. ****P < 0.0001 by 1-way ANOVA and Dunnett’s post hoc multiple-comparisons test.

Under steady-state conditions, adult *Mvk*^VI/Δ91^ mice did not show any differences in the frequencies of B cells, T cells, dendritic cells, neutrophils, or monocytes in peripheral blood, spleen, or BM (Supplemental Figure 4A) compared to wild-type mice or *Mvk^+/VI^* controls. Also, the level of inflammatory cytokines and chemokines in serum was barely detectable and did not differ between *Mvk*^VI/Δ91^ and *Mvk^+/VI^* animals (Supplemental Figure 4B). Similarly, serum IgD in wild-type and *Mvk*-mutant mice was below the limit of detection of a commercial ELISA (data not shown). The lack of inflammation under basal/unstimulated conditions was striking when compared with a proposed pharmacologic model of MKD (35), in which a bisphosphonate drug is administered i.p. to acutely inhibit protein prenylation (Figure 4A). Wild-type mice treated i.p. with the bisphosphonate alendronate, at comparable doses (6 mg/kg and 13 mg/kg) to those reported previously (35), showed a dramatic accumulation of uRab and uRap1A proteins in peritoneal cells 48 hours after treatment, a defect in prenylation that was far greater than in peritoneal cells from *Mvk*^VI/Δ91^ mice (Figure 4B). Importantly, while the frequencies of immune cell populations in the peritoneal cavity of *Mvk*^VI/Δ91^ mice were unchanged compared to controls (Figure 4, C and D), in wild-type animals alendronate treatment caused increased infiltration of neutrophils and monocytes, a trend toward increased eosinophils and small peritoneal macrophages, and a striking, dose-dependent decrease in large peritoneal macrophages (Figure 4, C and D). The higher dose of alendronate also appeared to reduce the proportion of Ly6C^hi^ monocytes compared with the lower dose (Figure 4, C and D).

### Mvk^VI/Δ91^ mice are hyperresponsive to endotoxin treatment and NLRP3 activation in vivo.

**Figure 5:**
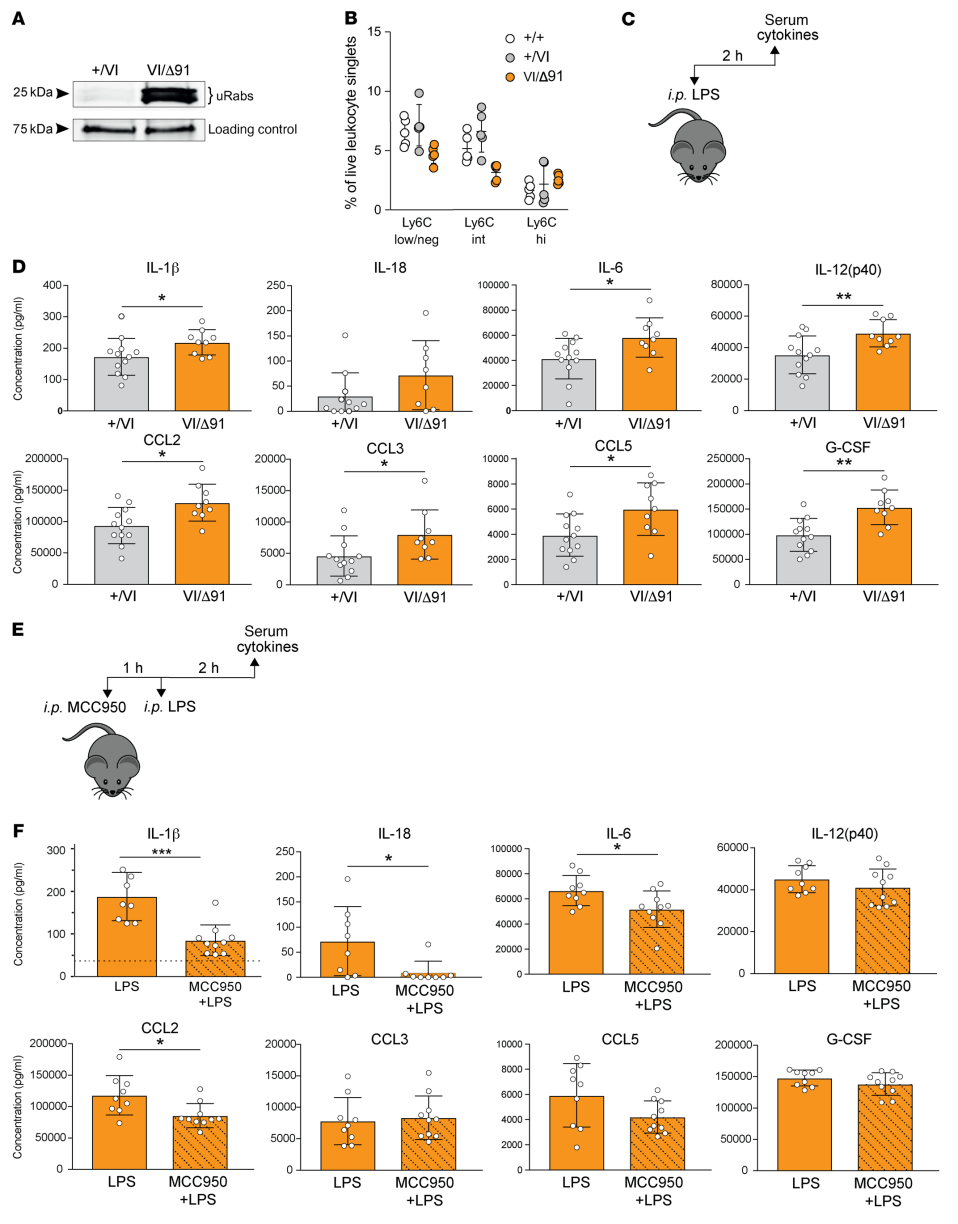
*Mvk*^VI/Δ91^ mice have elevated NLRP3-dependent IL-1β and other inflammatory mediators in serum following in vivo LPS treatment. (**A**) Detection of unprenylated Rab GTPases (uRabs) in PBMCs from *Mvk*^VI/Δ91^ mice compared to *Mvk^+/VI^* mice. (**B**) Flow cytometric analysis of monocyte populations in PBMCs from wild-type, *Mvk^+/VI^*, and *Mvk*^VI/Δ91^ mice. Monocytes were gated as live leukocyte singlets negative for B220, TCRβ, CD11c, and Ly6G, and with low/negative, intermediate, or high levels of Ly6C. Bars show the mean ± SD (5 mice per genotype) and are representative of 3 separate experiments. (**C**) *Mvk^+/VI^* mice (*n =* 12) and *Mvk*^VI/Δ91^ mice (*n =* 9) were administered i.p. LPS 2 hours before serum collection. (**D**) Serum cytokines and chemokines were measured using a multiplex assay or IL-18 ELISA. (**E**) *Mvk*^VI/Δ91^ mice were pretreated with i.p. 50 mg/kg MCC950 1 hour prior to LPS challenge (*n =* 9 with LPS alone, *n =* 10 with MCC950 + LPS). (**F**) Cytokines and chemokines were measured in serum 2 hours after the LPS challenge shown in **E**. The baseline level of serum IL-1β in untreated *Mvk*^VI/Δ91^ mice is indicated by a dotted line (see Supplemental Figure 5B for IL-1β). Other baseline values are shown in Supplemental Figure 4B and were too low to be represented on the same scale. In **D** and **F**, bars are mean ± SD. *P < 0.05; **P < 0.01; ***P < 0.001 by unpaired, 2-tailed Student’s *t* test with Welch’s correction. Each symbol represents a single mouse.

PBMCs from *Mvk*^VI/Δ91^ mice had a clear defect in protein prenylation, with marked accumulation of uRab proteins compared with PBMCs from control *Mvk^+/VI^* mice (Figure 5A and Supplemental Figure 3D). However, the proportion of circulating Ly6C^hi^ inflammatory monocytes was not significantly different between wild-type, *Mvk^+/VI^*, and *Mvk*^VI/Δ91^ mice (Figure 5B), and there were no differences in the expression of 754 myeloid innate immunity genes between freshly isolated PBMCs from *Mvk*^VI/Δ91^ and wild-type mice (Supplemental Figure 4C). Nonetheless, acute in vivo treatment of *Mvk*^VI/Δ91^ mice with i.p. LPS caused a significant increase in the levels of several inflammatory serum cytokines and chemokines (IL‑1β, IL‑18, IL‑6, G‑CSF, IL‑12, CCL2, CCL3, and CCL5) compared with control *Mvk^+/VI^* mice (Figure 5, C and D), with some variability between individual animals. Several of these factors (G-CSF, IL-6, IL-12, and CCL2) were also significantly elevated in the peritoneal fluid of *Mvk*^VI/Δ91^ mice compared with *Mvk^+/VI^* animals after in vivo LPS treatment (Supplemental Figure 5A). These same inflammatory factors (IL-1β, IL-18, IL-6, G‑CSF, IL-12, CCL2, CCL3, and CCL5) were also higher in serum from a patient with MKD, compared with healthy volunteers (Supplemental Figure 6). Surprisingly, the circulating levels of IFN-γ and TNF-α were not significantly different in LPS-treated *Mvk*^VI/Δ91^ mice, or in the MKD patient, compared with controls. In support of a role for NLRP3 in the enhanced in vivo inflammatory response, i.p. administration of a single dose of the NLRP3 inhibitor MCC950 (50 mg/kg) 1 hour prior to LPS challenge completely abolished the increase in IL‑18 and reduced IL-1β release to near-baseline circulating levels in *Mvk*^VI/Δ91^ mice (Figure 5, E and F, and Supplemental Figure 5B). Pretreatment with MCC950 also slightly, but significantly, reduced serum IL-6, CCL2, and CCL5, but not G-CSF, CCL3, or IL-12 (Figure 5F).

### Increased core body temperature exacerbates the defective mevalonate pathway in Mvk^VI/Δ91^ mice.

**Figure 6:**
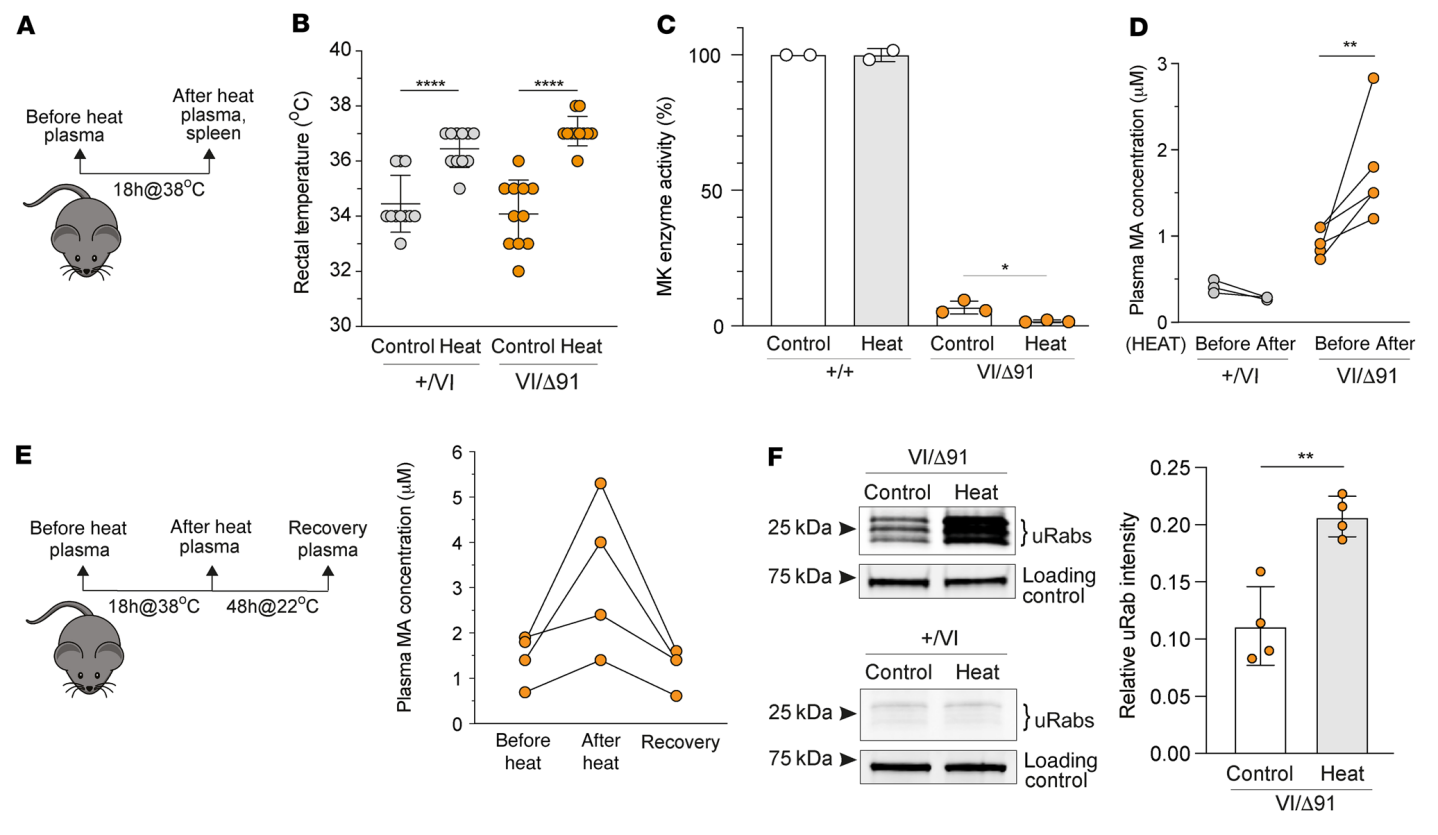
Elevated plasma mevalonic acid and defective protein prenylation are reversibly exacerbated in vivo in heated *Mvk*^VI/Δ91^ mice. (**A**) *Mvk^+/VI^* and *Mvk*^VI/Δ91^ mice were heated for 18 hours at 38°C. (**B**) Rectal temperature before and after heating. Bars show mean ± SD (*n =* 11 mice per group, each symbol represents a single mouse). ****P < 0.0001 by 1-way ANOVA with Tukey’s post hoc test. (**C**) Mevalonate kinase (MK) activity in splenocytes from unheated and heated wild-type and *Mvk*^VI/Δ91^ mice, expressed as a percentage of the MK activity in cells from an unheated wild-type mouse. Bars show mean ± SD, *n =* 2 wild-type mice and *n =* 3 *Mvk*^VI/Δ91^ mice per group. *P < 0.05 by unpaired, 2-tailed Student’s *t* test. (**D**) Levels of plasma mevalonic acid (MA) before and after heating (*n =* 3 *Mvk^+/VI^* mice, *n =* 5 *Mvk*^VI/Δ91^ mice). **P < 0.01 by 1-way ANOVA with Tukey’s post hoc test. (**E**) Experimental design for heat exposure of mice, followed by 48 hours’ recovery. Data show plasma MA concentration in 4 *Mvk*^VI/Δ91^ mice measured before, after, and after 48 hours’ recovery from heating. (**F**) Unprenylated Rab GTPases (uRabs) in *Mvk*^VI/Δ91^ and *Mvk^+/VI^* splenocytes from heated and unheated mice (representative blots from 4 mice per group). For *Mvk*^VI/Δ91^ samples, blots were analyzed by densitometry and values of uRab intensity were normalized to the loading control. Bars show mean ± SD, *n =* 4 mice. **P < 0.01 by unpaired, 2-tailed Student’s *t* test with Welch’s correction. Each symbol represents a single mouse.

Mutations in the human MK protein may render the enzyme more sensitive to increased temperature (8, 28), thereby potentially exacerbating the defect in protein prenylation in MKD. To test this hypothesis in vivo, we took advantage of the fact that homeostasis in mice becomes dysregulated at ambient temperatures above 30°C (44). To increase core body temperature (T_core_) we transferred cages of mice from standard housing temperature (22°C ± 1°C) to a heated chamber at 38°C for 18 hours (Figure 6A). This procedure increased T_core_ in *Mvk^+/VI^* and *Mvk*^VI/Δ91^ mice by 2.5°C (from approximately 34.5°C to 37°C; Figure 6B), as measured using a digital rectal temperature probe.

MK activity was 3-fold lower (and barely detectable) in spleen cells from heated *Mvk*^VI/Δ91^ mice compared with unheated mice, but was unaltered in spleen cells from heated and unheated control *Mvk^+/+^* animals (Figure 6C). Similarly, increased temperature did not affect plasma MA in wild-type mice, but in *Mvk*^VI/Δ91^ animals heating resulted in a significant further elevation in circulating MA (Figure 6D), which was accompanied by a worsening of the defect in Rab prenylation (Figure 6F and Supplemental Figure 7A). Importantly, plasma MA returned to baseline levels in *Mvk*^VI/Δ91^ animals upon 48 hours’ recovery at 22°C (Figure 6E). Despite the enhanced loss of prenylation in heated *Mvk*^VI/Δ91^ mice, there was no indication (Supplemental Figure 7B) of the cell infiltration and peritoneal inflammation observed in wild-type mice treated with alendronate (Figure 4).

### Prenylation in cells from mice and humans with MKD is sensitive to increased temperature.

**Figure 7:**
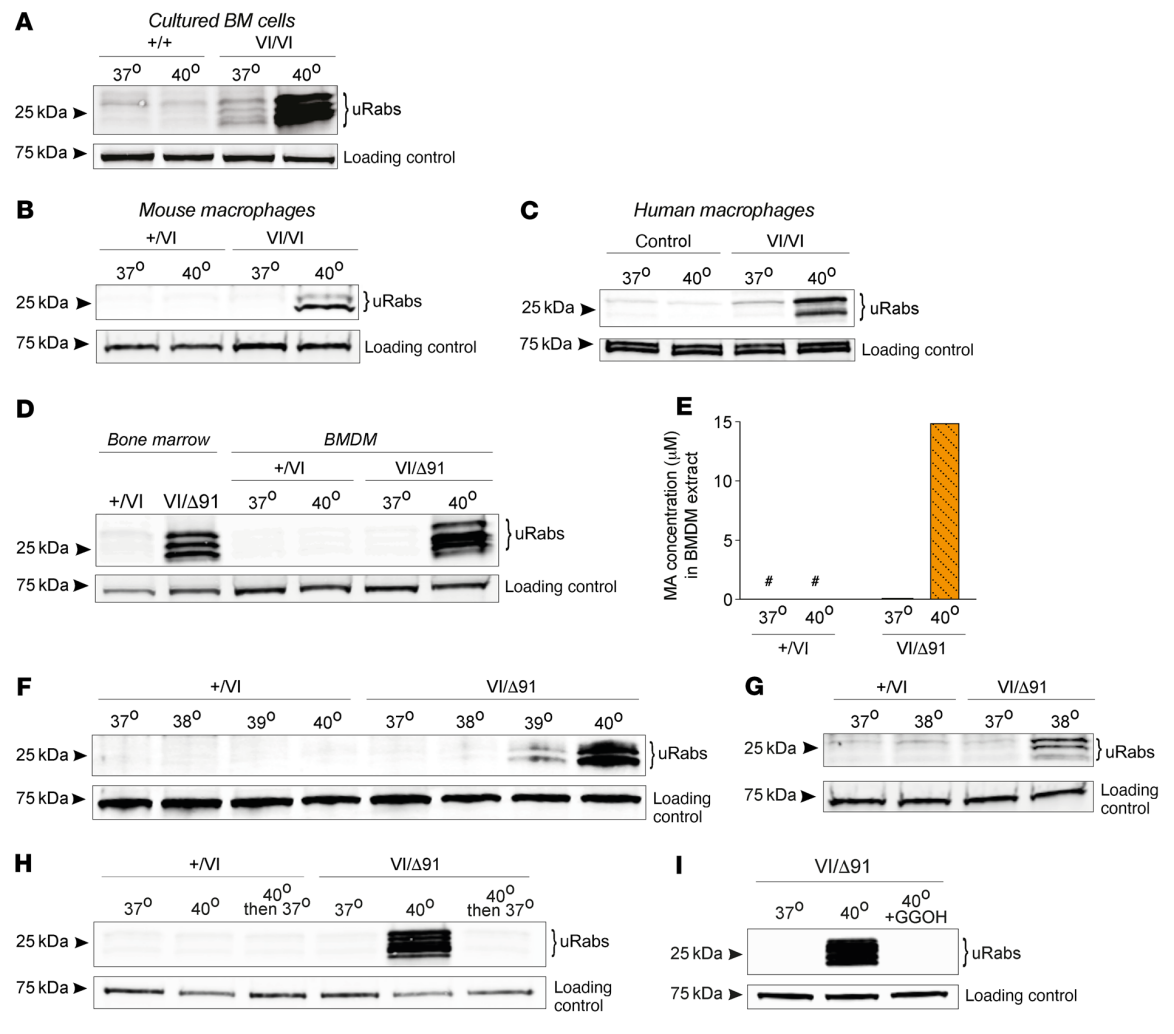
Increases in temperature enhance defective protein prenylation in mouse and human MKD cells. Mouse and human cells were cultured at 37°C and 40°C for 24 hours (unless otherwise specified) and analyzed for the presence of unprenylated Rabs (uRabs). (**A**) Bone marrow (BM) cells from wild-type and *Mvk^VI/VI^* mice. (**B**) BMDMs from *Mvk^+/VI^* and *Mvk^VI/VI^* mice. (**C**) Human monocyte-derived macrophages from a healthy control and *MVK^V377I/V377I^* (VI/VI) patient. (**D**) Comparison of fresh whole bone marrow and cultured BMDMs from *Mvk^+/VI^* and *Mvk*^VI/Δ91^ mice. (**E**) Concentration of mevalonic acid (MA) in *Mvk^+/VI^* and *Mvk*^VI/Δ91^ BMDMs (# = below limit of detection). Comparison of uRabs in *Mvk^+/VI^* and *Mvk*^VI/Δ91^ BMDMs cultured (**F**) at 37°C or 40°C for 24 hours, (**G**) at 37°or 38°C for 5 days, and (**H**) at 37°C or 40°C for 24 hours, and at 40°C followed by 24 hours recovery at 37°C. (**I**) uRabs in *Mvk*^VI/Δ91^ BMDMs cultured for 24 hours at 37°C or 40°C in the absence or presence of 10 μM GGOH. Blots shown in **A**, **B**, **D**, and **F**–**I** are representative of 3 independent experiments.

We further examined the effect of heat on *Mvk*-mutant mouse cells by culturing freshly isolated BM cells and BM-derived macrophages (BMDMs) at either 37°C or 40°C for 24 hours. Higher temperatures did not affect prenylation in wild-type BM (Figure 7A). In contrast, the mild accumulation of uRabs in *Mvk^VI/VI^* BM cells was dramatically increased when cultured at 40°C (Figure 7A). Interestingly, unlike *Mvk^VI/VI^* BM, *Mvk^VI/VI^* BMDMs had no detectable accumulation of uRab proteins at 37°C but had a clear prenylation defect when cultured at 40°C (Figure 7B). A remarkably similar effect was observed when PBMCs from a HIDS patient of the same genotype (*MVK^V377I/V377I^*) were cultured at 40°C (Figure 7C).

Like *Mvk^VI/VI^* BMDMs, *Mvk*^VI/Δ91^ BMDMs had no detectable defect in protein prenylation compared to freshly isolated BM. However, culturing *Mvk*^VI/Δ91^ BMDMs at 40°C for 24 hours resulted in a clear accumulation of uRabs (Figure 7D), and a 50-fold increase in intracellular MA (Figure 7E). Even a 2°C temperature increase (39°C) for 24 hours was sufficient to cause the accumulation of uRabs in *Mvk*^VI/Δ91^ BMDMs (Figure 7F), whereas a 5-day period was needed to have a detectable effect at 38°C (Figure 7G). Increased temperature had no effect on Rab prenylation or MA levels in control *Mvk^+/VI^* BMDMs (Figure 7, B, D, and F–H).

Importantly, the dramatic buildup of uRab proteins in heated (40°C) *Mvk*^VI/Δ91^ BMDMs was lost after 24 hours’ recovery at 37°C (Figure 7H), likely due to partial restoration of enzyme activity and/or degradation of unprenylated proteins that accumulated during heat exposure.

Remarkably, the dramatic appearance of uRabs in heated *Mvk*^VI/Δ91^ BMDMs was completely abolished in the presence of 10 μM geranylgeraniol (GGOH) (Figure 7I), an isoprenoid lipid precursor that can serve as substrate for protein prenylation when endogenous levels of GGPP are depleted.

## Discussion

How inflammatory flares are triggered in MKD remains unclear, largely due to the lack of genetic mouse models carrying *Mvk* mutations analogous to those in MKD patients. We show here that mice bearing combinations of hypomorphic (p.V377I) and amorphic (frameshift) mutations in *Mvk* recapitulate the characteristic biochemical features of HIDS, the milder form of MKD. Humans heterozygous for pathogenic *MVK* variants have reduced MK enzyme activity (3, 45, 46) but lack any detectable defect in protein prenylation (19, 20). Similarly, heterozygous *Mvk*-mutant mice showed normal prenylation of small GTPases despite a 25% to 50% decrease in MK activity. However, humans and mice homozygous for p.V377I (abbreviated as *Mvk^VI^*), the most frequent pathogenic variant in HIDS (5, 9, 10, 27), had a mild defect in protein prenylation. In contrast, compound heterozygous *Mvk*^VI/Δ91^ mice (lacking 1 functional allele) had a pronounced prenylation defect, comparable to that of a compound heterozygous HIDS patient bearing the more severe p.H20N mutation on 1 allele (19, 20).

The extent of the defect in prenylation in *Mvk*^VI/Δ91^ and *Mvk^VI/VI^* mice and their residual MK activity (9% and 19%, respectively) suggests that these mutant mice represent opposite biochemical ends of the milder (HIDS) spectrum of MKD. Furthermore, by comparing the residual MK enzyme activity in heterozygous, homozygous, and compound heterozygous mice with the extent of their prenylation defect, we found a distinct boundary around 20% MK activity. Above this threshold, prenylation can be maintained normally (as in *Mvk*^+/Δ91^ and *Mvk^+/VI^* mice with >40% MK activity). However, less than 20% enzyme activity leads to insufficient synthesis of the lipid substrate GGPP and loss of prenylation (as in *Mvk*^VI/Δ91^ mice with 9% activity). This is entirely consistent with measurements of residual MK activities below 20% reported in compound heterozygous individuals with HIDS (11). *Mvk^VI/VI^* mice lie on this precarious border, with 19% enzyme activity and a very mild prenylation phenotype. Humans homozygous for *MVK^V377I^* probably also lie on this threshold, thus explaining why some individuals homozygous for this variant are either unaffected or generally mildly symptomatic (9, 47). The ability of 1 *Mvk^VI^* allele to confer about 10% residual MK activity in mice also explains why the *MVK^V377I^* mutation is not associated with mevalonic aciduria in humans (6, 10), in which MK enzyme activity is consistently less than 0.5% (13–15). We show that 1 *Mvk^VI^* allele confers approximately 10% MK activity, enough to avert mevalonic aciduria even if the second *Mvk* allele bears a complete loss-of-function mutation (as in *Mvk*^VI/Δ91^ mice).

In the p.V377I variant of the MK enzyme, the conservative Val>Ile substitution is distant from the active site, and thus its impact on MK activity has remained obscure (38). Using Aquaria to visualize the 3D structure of MK, we demonstrate that this residue lies within a short, highly conserved segment near the C-terminus (residues 375–377) that forms part of the hydrophobic core of the enzyme. In support of this, variants affecting the neighboring amino acid Gly376 have also been reported in patients with MKD in the online INFEVERS database (48). Any alterations to this short segment, including p.V377I, are therefore likely to disrupt the critical core structure of the MK enzyme.

Our finding that the level of residual MK activity in vivo determines the extent of the defect in protein prenylation calls into question the physiological relevance of other proposed models of MKD in which the capacity for normal prenylation is far more severely compromised. These include conditional loss of geranylgeranyltransferase I (24), or acute inhibition of prenylation in vivo by i.p. administration of the bisphosphonate alendronate (33, 35). We found that alendronate treatment in wild-type mice, at the doses reported previously (33, 35), caused a far greater effect on prenylation in peritoneal cells than the endogenous prenylation defect in *Mvk*^VI/Δ91^ mice, but also triggered a local inflammatory response that was absent in *Mvk*-mutant mice. Alendronate-induced inflammation was clearly revealed by the influx of neutrophils and Ly6C^hi^ monocytes, and the loss of large peritoneal macrophages after alendronate treatment is consistent with the inflammation-induced macrophage disappearance reaction (49). Increased cell death, a well-described effect of bisphosphonate drugs that inhibit prenylation (30, 50), is the most likely cause of alendronate-induced inflammation in the peritoneal cavity. The absence of detectable signs of inflammation in *Mvk*^VI/Δ91^ mice under steady-state conditions is consistent with the fact that HIDS patients can be healthy between flares. Importantly, it also highlights the critical role of additional triggers (such as increased temperature, discussed below) that may tip the homeostatic balance toward exacerbating the biochemical deficiency in MK.

As well as recapitulating the prenylation phenotype of HIDS, the *Mvk*-mutant mice described here also showed changes in plasma MA that are consistent with defective MK activity. Elevated plasma and urinary MA is a characteristic feature of MKD first described in patients with mevalonic aciduria (13, 14). However, urinary MA is also mildly raised in HIDS during flares (3–5, 15). We found that, unlike the milder *Mvk^VI/VI^* genotype, *Mvk*^VI/Δ91^ mice had slightly but significantly elevated MA in plasma and BM cell extracts even under steady-state conditions, and this is consistent with their lower residual MK activity and greater defect in prenylation. *Mvk*-mutant mice did not have elevated serum IgD. However, this feature is not considered to be a specific or reliable indication of HIDS/MKD in humans (51) and may be secondary to chronic inflammation (3, 5, 52).

The main source of MA in plasma is likely to be nonhemopoietic tissue, because the transfer of *Mvk*^VI/Δ91^ BM into wild-type animals had little effect on the levels of circulating MA. Furthermore, plasma MA levels remained high in *Mvk*^VI/Δ91^ animals even after replacement with wild-type BM. This is entirely consistent with reports that severe MKD patients that underwent successful hematopoietic stem cell transplantation (HSCT) maintained persistently elevated levels of urinary MA despite alleviation of fever and inflammatory symptoms (45, 46). Hence, urinary and/or plasma MA levels are not appropriate indicators of the success of HSCT therapy in MKD patients, reinforcing the need for careful monitoring of disease recurrence (46). Liver, the main site of mevalonate-cholesterol biosynthesis (53), is likely the major source of the elevated plasma MA in mice and humans with MKD, at least under steady-state/nonfebrile conditions. This also explains why treatment with simvastatin, a drug that selectively targets liver, decreased MA levels in HIDS patients (54).

It has recently been suggested that MA can train innate immune cells to respond more robustly to stimulation via increased histone acetylation and epigenetic remodeling (55). We do not favor this as a physiological cause of inflammation in MKD because the concentration of MA used to train mouse macrophages in vitro, 500 μM (55), is 100 times higher than the plasma level of MA we detected in *Mvk*^VI/Δ91^ mice, and measurements of plasma MA even in mevalonic aciduria patients are mostly below 150 μM (14). Furthermore, statin therapy (which blocks MA synthesis) is considered not to be generally beneficial in the treatment of MKD (56), did not reduce the severity, frequency, or occurrence of flares despite lowering urinary MA (54), and even caused severe clinical crisis in 2 mevalonic aciduria patients (14). Finally, MA levels remained persistently elevated in several MKD patients after HSCT despite the alleviation of fever and inflammatory symptoms (45, 46). Together, these observations strongly suggest that MA accumulation is not the main underlying cause of inflammation in MKD. However, MA (or its derivative mevalonolactone) may contribute to the neurological features of mevalonic aciduria (57). Indeed, neurological function in a patient with mevalonic aciduria substantially improved after liver transplant but inflammatory episodes only resolved after HSCT (58).

Lack of normal protein prenylation is the most likely cause of inflammation in MKD. We recently provided direct evidence that protein prenylation is defective in freshly isolated cells from individuals with MKD (19, 20). Decreased prenylation (specifically, geranylgeranylation) of small GTPases such as Rac leads to enhanced inflammasome formation and subsequent increase in the processing and release of IL-1β (22, 23, 32, 36, 59). IL-1β release after LPS stimulation was enhanced in *Mvk*^VI/Δ91^ mice and dependent on the NLRP3 inflammasome, since it was blocked by treatment with the specific NLRP3 inhibitor MCC950 (60, 61). These findings are consistent with our recent report that increased IL-1β production from LPS-stimulated patient PBMCs is abolished by MCC950 (23). Thus, inhibition of the NLRP3 inflammasome may be an appropriate therapeutic approach for MKD. The pyrin inflammasome may also play a role in MKD, although evidence for this is based on in vitro studies using pharmacological inhibitors or cells completely deficient in geranylgeranyltransferase I activity (24, 25), which may not be valid physiological models of MKD. Cytokines other than IL-1β may also be important in MKD pathology, with evidence for increased levels of a variety of proinflammatory cytokines from MKD PBMCs after stimulation (62). We also found significantly higher concentrations of various serum cytokines and chemokines in LPS-treated *Mvk*^VI/Δ91^ mice, not all of which were reduced by MCC950 treatment. The inflammatory flares in MKD are therefore likely to be a complex, multiple-cytokine-driven process. This is consistent with the fact that IL-1β–neutralizing treatment is effective in some but not all MKD patients (56, 63, 64), and therapies targeting IL-6 and TNF-α can also be beneficial (64). The exact mechanisms by which deficient protein prenylation in MKD leads to enhanced NLRP3 inflammasome activation remain to be determined, but may involve reactive oxygen species (23).

Infection, vaccinations, strenuous exercise, and psychological stress are recognized as triggers of inflammatory flares in individuals with MKD (5, 12, 26, 27, 56). Notably, all of these can cause an increase in T_core_. Infection and vaccination can induce a PGE_2_-dependent rise in T_core_ (65, 66), while acute and chronic stress can also elicit an increase in T_core_ via an autonomic response known as psychological stress–induced hyperthermia (67, 68). Higher temperatures have been suggested to exacerbate the abnormal folding/stability of mutant MK protein (3, 5, 28, 38, 69). Consistent with this, we found that *Mvk^VI/VI^* and *Mvk*^VI/Δ91^ cells showed a striking sensitivity to elevated temperature, similar to MKD lymphoblast cell lines (19, 36). Even a 1°C increase in temperature for a few days was sufficient to enhance the prenylation defect in cultured *Mvk*^VI/Δ91^ macrophages. A 3°C rise, equivalent to the high fever that is common in MKD patients (12), caused a rapid and dramatic buildup of unprenylated proteins in *Mvk*^VI/Δ91^ and *Mvk^VI/VI^* cells, as well as human *MVK^VI/VI^* macrophages. This effect was clearly reversible in *Mvk*^VI/Δ91^ BMDMs when the cells were returned to 37°C.

Most importantly, elevated temperature also worsened the metabolic defect in *Mvk*^VI/Δ91^ mice. Increasing T_core_ by 2°C to 3°C further compromised the residual MK activity in splenocytes from 9% to less than 2%, and led to even higher levels of plasma MA and clear exacerbation of the defect in protein prenylation. Strikingly, and like in cultured BMDMs, this phenomenon was reversible and plasma MA returned to baseline levels once T_core_ was restored. This is reminiscent of the spike in urinary MA observed in HIDS patients during fever-associated flares (3, 4, 12, 15). Although the increase in plasma MA could reflect altered blood osmolality caused by dehydration in heated mice, this does not explain the dramatic buildup of intracellular MA that we also observed in cultured *Mvk*^VI/Δ91^ macrophages after heating. Together, these findings provide compelling evidence that elevated T_core_ could trigger inflammatory flares in MKD by temporarily exacerbating the underlying defect in MK activity and protein prenylation, an idea proposed by Houten and colleagues 20 years ago (28) but not formally tested. Approaches to minimize increases in T_core_ by pharmacological modulation of the central thermal regulatory network (66) could therefore be considered as a future strategy to manage MKD. In addition, replenishment of isoprenoid lipid precursors should also be considered as an approach to prevent the worsening of defective prenylation in response to elevated T_core._ GGOH is a cell-permeant analog of GGPP that can overcome a lack of this isoprenoid lipid (23, 70). We show that replenishing *Mvk*^VI/Δ91^ BMDMs with GGOH completely prevented the dramatic intracellular buildup of uRabs in response to heat. *Mvk*-mutant mice will therefore provide a much-needed preclinical platform to test the bioavailability, effectiveness, and safety of GGOH or other lipid supplements as a preventive treatment in MKD.

The striking loss of the prenylation defect in cultured *Mvk^VI/VI^* and *Mvk*^VI/Δ91^ BMDMs compared with fresh BM cells confirms reports that cells obtained or derived from MKD patients appear to adapt in culture (71). Furthermore, the ability of heat to cause defective prenylation in *Mvk*-mutant BMDMs is consistent with our previous observation that heat triggers loss of protein prenylation in MKD patient–derived cell lines (19, 36). This explains why abnormal prenylation has proven so difficult to demonstrate in cultured MKD cells (71), because they likely adapt by upregulating MK and other enzymes of the mevalonate pathway (36, 71–73). Furthermore, infection of B lymphocytes with Epstein-Barr virus (EBV, which is routinely used to create immortalized lymphoblast cell lines from patients) causes upregulation of the mevalonate pathway (74). For this reason, accurate measurements of residual MK activity should ideally be performed with freshly isolated PBMCs, rather than with cultured cells or EBV-transformed cell lines.

**Figure 8:**
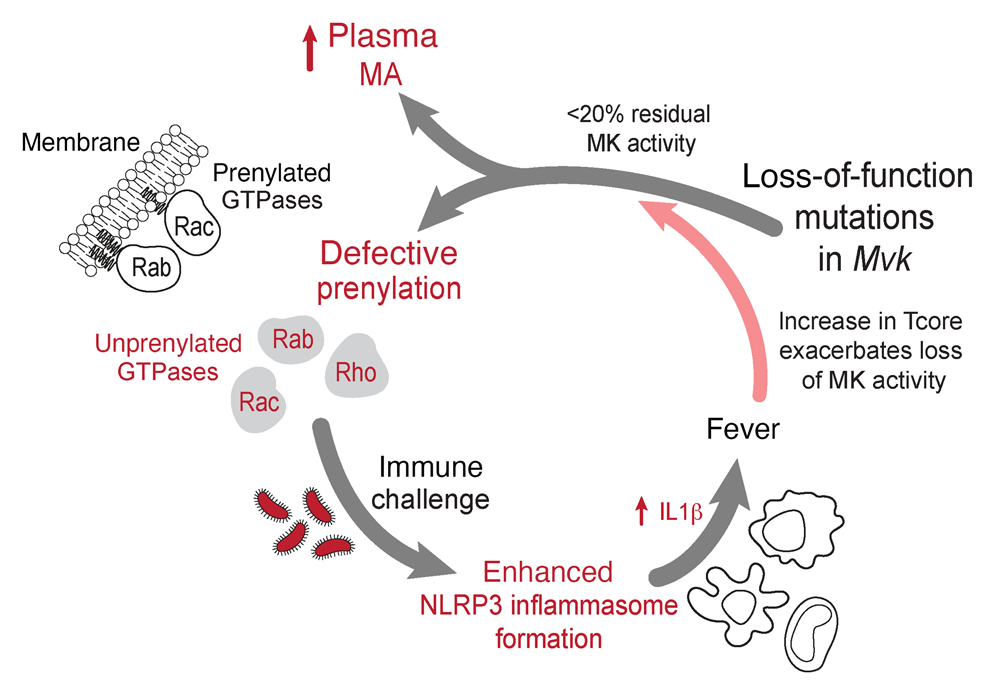
Pathophysiological features of inflammatory flares in MKD — lessons from mouse models. Genetic variants in *Mvk* causing <20% residual mevalonate kinase (MK) activity lead to a buildup of plasma mevalonic acid (MA, probably derived mostly from liver) and intracellular accumulation of unprenylated small GTPase proteins. The latter enhance NLRP3 inflammasome formation after immune challenge, causing excessive release of IL-1β and other proinflammatory cytokines and chemokines. Elevated core body temperature (i.e., fever due to infection, vaccination, or stress) exacerbates the defect in mutant MK enzymes, further worsening the metabolic deficiency in the mevalonate pathway and triggering an inflammatory flare.

In summary, we show that mouse models of MKD, created by CRISPR/Cas9 editing of the *Mvk* gene, recapitulate the biochemical and clinical diagnostic features of the human disease. Our findings demonstrate a role for the NLRP3 inflammasome in the enhanced production of proinflammatory IL-1β and IL-18 in vivo. Importantly, we demonstrate a mechanism whereby increased T_core_ (e.g., in response to infection, stress, or vaccination) exacerbates the deficiency in protein prenylation in MKD, thus triggering inflammatory flares that resolve after T_core_ returns to normal (Figure 8). These animal models of MKD will continue to shed further light on the pathophysiology of the disease and constitute a valuable preclinical resource for testing new therapeutic approaches to overcome the metabolic defect and prevent inflammatory flares.

## Methods

### Generation of Mvk-mutant mice.

*Mvk*-mutant mice were produced by the Mouse Engineering Garvan/ABR (MEGA) Facility using CRISPR/Cas9 gene targeting in C57BL/6J mouse embryos following established molecular and animal husbandry techniques (75). The single guide RNA (sgRNA) was based on a target site in exon 11 of *Mvk* (AGCTGAGTGTGTGGAAACTCCGG, where CGG = protospacer-associated motif/PAM, underlined) and was microinjected into the nucleus and cytoplasm of C57BL/6J zygotes together with polyadenylated *Streptococcus* *pyogenes* Cas9 mRNA and a 140 base single-stranded, antisense, deoxyoligonucleotide homologous recombination substrate carrying the p.Val377Ile (GTT>ATT) mutation and a PAM-inactivating silent mutation in the P375 codon (CCC>CCA). A male founder mouse heterozygous for both substitutions was then backcrossed with C57BL/6J female mice to establish the heterozygous *Mvk^V377I^* line (referred to as *Mvk^+/VI^*). Three independent heterozygous *Mvk*^Δ^ lines were generated carrying deletions in exon 11 (*Mvk*^+/Δ8^, *Mvk*^+/Δ13^, *Mvk*^+/Δ91^) resulting in frameshift mutations following the codons for T370, A374, and G347, respectively. Mice were crossed to generate homozygous *Mvk^VI/VI^* and compound heterozygous *Mvk*^VI/Δ8^, *Mvk*^VI/Δ13^, and *Mvk*^VI/Δ91^ mice. Animals were bred and housed with standard chow diet in specific pathogen–free conditions at Australian BioResources and the Garvan Institute Biological Testing Facility and used at 10 to 12 weeks of age.

### BM chimeras.

Recipient female *Mvk*^VI/Δ91^ (*Ptprc^b^*) and B6.SJL-*Ptprc^a^Pepc^b^*/BoyJArc mice (Australian Resources Centre), age 8 to 9 weeks, were irradiated twice with 4.25 Gy administered 6 hours apart and injected intravenously with 5 × 10^6^ to 10 × 10^6^ BM cells from female B6.SJL-*Ptprc^a^Pepc^b^*/BoyJArc or *Mvk*^VI/Δ91^ donor mice. Eight weeks later, plasma samples from chimeric mice were analyzed for MA as described below.

### Immune cell isolation.

Whole BM was flushed from the femur and tibia and erythrocytes were lysed by incubation in 0.83% NH_4_Cl, 0.1% KHCO_3_ for 5 minutes at room temperature, and then washed in cold Mg^2+^- and Ca^2+^-free DPBS (Gibco). PBMCs were isolated from age- and sex-matched mice by centrifugation over Ficoll-Paque Plus (GE Healthcare). Splenocytes were obtained by crushing the spleen through a 70 μm nylon filter (Falcon), followed by erythrocyte lysis as above. Peritoneal cells were harvested by lavaging the peritoneal cavity with 5 mL cold 2 mM EDTA/Mg^2+^- and Ca^2+^-free PBS (GIBCO). Isolated cells were then used for RNA extraction, lysate preparation, or stained with conjugated antibody for flow cytometry.

### PBMCs and monocyte-derived macrophages from MKD patients.

Fresh blood samples were obtained from healthy volunteers and from MKD (HIDS) patients with confirmed pathogenic *MVK* variants — adult males homozygous for p.V377I or compound heterozygous for p.V377I/p.Tyr149_Ser150insAlaTyr (20), and a male child compound heterozygous for p.V377I/p.H20N (19, 20). Patients were between flares when samples were collected. Buffy coat preparations of PBMCs were isolated by centrifugation over Ficoll-Paque Plus. PBMC pellets were snap frozen for analysis of unprenylated proteins.

Human macrophages were generated from a 20-year-old female with MKD (homozygous for p.V377I), and an age- and sex-matched healthy donor, by culturing PBMCs in RPMI supplemented with 10% heat-inactivated fetal calf serum (FCS), 50 U/mL penicillin, 50 μg/mL streptomycin (GIBCO), and 100 ng/mL rhM-CSF (Sino Biologicals) on untreated tissue culture plates in a humidified incubator (5% CO_2_) at 37°C for 7–10 days, replacing half the volume with fresh culture medium every 2 days. The resulting macrophages were incubated either at 37°C or 40°C for 24 hours before collection. Cells pellets were analyzed for the presence of uRab proteins as described below.

### BMDMs.

BMDMs were generated by culturing BM, flushed from femurs and tibias, in culture medium (RPMI, 10% heat-inactivated FCS, 50 U/mL penicillin, 50 μg/mL streptomycin) supplemented with 50 ng/mL rhM‑CSF on untreated plates in a humidified incubator with 5% CO_2_ at 37°C for 4 days. Adherent cells were then replated and cultured for 24 hours at 37°C, 38°C, 39°C, or 40°C, or at 38°C for 5 days, before harvesting. To test the effect of GGOH (Sigma-Aldrich), a 10 mM stock solution in ethanol was added to the culture medium at a final concentration of 10 μM. Cell pellets were analyzed for the presence of uRab proteins or MA.

### Detection of unprenylated proteins and Western blotting.

An in vitro prenylation assay was used to measure defective protein prenylation (19, 36, 37). Briefly, uRab proteins in cell lysates were prenylated by incubation with recombinant Rab geranylgeranyltransferase and a synthetic biotin-isoprenoid lipid substrate, and detected on PVDF membranes using streptavidin-680RD (LI-COR) (37).

Some lysates were also analyzed by Western blotting for the presence of uRap1A using anti-Rap1A (Santa Cruz Biotechnology, sc-1482) with anti-goat 680RD secondary antibody (LI-COR) (30, 37). β-Actin (Cell Signaling Technology, 3700) or a narrow doublet of endogenous biotinylated protein approximately 73 kDa (often appearing as a broad singlet) was used as a sample loading control (37). Blots were scanned on a LI‑COR Odyssey imager and analyzed using Image Studio v5.2.5. MK protein was detected in liver homogenates by Western blotting using 1 μg/mL rabbit polyclonal antibody (Antibodies Online) against amino acids 179–228 of human MK, and HRP-conjugated anti-rabbit IgG. Blots were developed using SuperSignal West Pico reagent (Thermo Fisher Scientific) and scanned on a Fusion FX7 imaging system (Etablissements Vilber Lourmat SAS). Densitometry was performed using ImageJ v2.0.0 (NIH).

### LC-MS/MS analysis of MA.

For analysis of MA in cells, 1 mL cold extraction solvent (80:20 methanol/water) was added to frozen cell pellets and then vortexed for 10 seconds and incubated in an ultrasonic bath filled with ice water for 1 hour, and then centrifuged at 1811*g* for 30 minutes at 4°C. Aliquots (850 μL) of supernatant were dried under vacuum in an Eppendorf Concentrator Plus and then reconstituted in 42.5 μL 70% methanol, 30% 10 mM ammonium acetate. Samples (injection volume 1 μL) were analyzed by targeted LC-MS/MS using an Agilent 1290 Infinity II UHPLC system coupled to an Agilent 6495 triple quadrupole mass spectrometer. Separation was achieved using an Agilent Infinity Poroshell 120 EC-C18 column (3.0 × 150 mm, 2.7 μm) fitted with an Agilent Infinity Poroshell 120 EC-C18 UHPLC guard column (3.0 × 5 mm, 2.7 μm), maintained at 20°C. The mobile phases were 10 mM ammonium acetate in water (A) and methanol (B), both containing 5 μM medronic acid to chelate metal ions (gradient 98% A from 0 to 3 minutes, decreased to 2% A from 3.5 to 6.5 minutes at 0.5 mL/min, then increased to 98% A at 0.4 mL/min from 6.5 to 12 minutes; total run time 12 minutes). Autosampler temperature was 4°C. The mass spectrometer was operated in negative electrospray ionization mode; source gas temperature was 250°C with flow at 17 L/min, sheath gas temperature was 400°C with flow at 12 L/min, and nebulizer pressure was 45 psi. Data were acquired in MRM (multiple reaction monitoring) mode and were processed using Agilent MassHunter Quantitative Analysis software version B08.00.00. By comparison with a pure standard (Sigma-Aldrich), MA eluted at a retention time of 1.9 minutes. The limit of detection was 0.1 μM.

### Measurement of MK activity.

Immediately after culling, liver was perfused with saline via the portal vein and snap-frozen. Slices of frozen liver, or frozen pellets of splenocytes and BM, were homogenized in buffer (1:2.5 weight/volume for liver) containing 100 mM KPO_4_ pH 7.4, 5 mM MgCl_2_, 1mM DTT, and 1× Roche complete EDTA-free protease inhibitor cocktail, using an ice-cold dounce homogenizer. After centrifuging (10,000*g* for 30 minutes, 4°C), MK activity in the supernatant was measured using a modification of previously described methods (76, 77). Briefly, 80 μg of homogenate was added to a total volume of 23 μL reaction buffer: 100 mM KPO_4_ pH 7.4, 6 mM MgCl_2_, 4 mM ATP, with 0.33 mM *R*,*S*-[2-^14^C]mevalonic acid (53 mCi/mmol; PerkinElmer) hydrolyzed from the lactone form. After incubation at 37°C for 30 minutes, 1.7 μL 88% formic acid was added and then left for 30 minutes at room temperature. Supernatant (10 μL) was spotted in duplicate onto Cellulose F thin-layer chromatography plates (Millipore) and developed in *n*-butanol/formic acid/water (77:10:13) for 5 hours. Dried plates were exposed to a phosphorscreen and imaged using a FujiFilm Typhoon FLA5100 scanner. Mevalonate-5-phosphate and unreacted mevalonolactone were identified with *R_f_* values of 0.19 and 0.78 respectively, consistent with previous studies (77). Densitometry to determine mevalonate-5-phosphate was performed using LI-COR Image Studio v5.2.5 and MK activity was calculated as percentage of wild-type control.

### MK sequence and structure analysis.

To examine all 3D structures related to mouse MK, we used Aquaria (39), which is based on sequence-to-structure alignments generated by HHblits (41). We also used the Aquaria interface to assign CATH domains (43) and ConSurf conservation scores (78), with the latter fetched from PredictProtein (79). PredictProtein/ConSurf assigned each residue to have low, intermediate, or high conservation, based on a Bayesian analysis (80) of evolutionary relatedness between the query protein and homologs in UniProt (The UniProt Consortium 2019). To create the figures showing structures mapped with domains, conservation, rat versus mouse sequence similarity, as well as the V377I and Δ91 variants, we used a version of Aquaria currently in development (39, 40) and available at https://aquaria.app/

### Alendronate treatment in vivo.

A stock solution of 2.6 mg/mL alendronate sodium salt (Sigma-Aldrich) was prepared by dissolving in saline and adjusted to pH 7.4. Mice were injected i.p. with alendronate (6 mg/kg, 13 mg/kg) or saline alone, and peritoneal cells were harvested 48 hours later by lavaging the peritoneal cavity with 5 mL cold 2 mM EDTA/ Mg^2+^- and Ca^2+^-free PBS. Peritoneal cells were analyzed by flow cytometry, or pooled from 3 mice for analysis of unprenylated proteins.

### Flow cytometry.

Cells from BM, spleen, blood, and peritoneal cavity were collected as described above and cell viability and total cell numbers were assessed by trypan blue staining using a Corning CytoSMART cell counter. Cells were preincubated with mouse-Fc block and viability dye (Zombie Aqua, BioLegend) in Mg^2+^- and Ca^2+^-free PBS for 15 minutes, before staining with fluorescently conjugated antibodies (Supplemental Table 1) prepared in washing buffer (WB: 2 mM EDTA, 0.02% sodium azide; 0.5% FCS, calcium/magnesium-free PBS) for 45 minutes. Samples were rinsed 3 times in WB and fixed with 10% formalin neutral buffered solution (Sigma-Aldrich), washed, and resuspended in WB for processing with a BD LSRII SORP flow cytometer/DIVA software. Postacquisition analysis was performed using FlowJo 10.6.2 (BD).

### Gene expression analysis.

Blood was collected and pooled from wild-type or *Mvk*^VI/Δ91^ female mice (*n =* 4 mice per genotype). PBMCs were isolated by centrifugation over Ficoll-Paque Plus (GE Healthcare) in SepMate tubes (STEMCELL Technologies) and then RNA was extracted using TRI reagent (Sigma-Aldrich, T9424). RNA (50 ng) was hybridized at 65°C overnight according to the manufacturer’s instructions in a thermocycler using the nCounter Mouse Myeloid Innate Immunity Panel V2 (Nanostring Technologies). Hybridized samples were purified and immobilized onto a sample cartridge using the nCounter Prep Station and then analyzed using the nCounter Digital Analyzer. Raw mRNA abundance frequencies were normalized to housekeeping and positive control genes using the nSolver Analysis Software 4.0. Values are expressed as log_2_ counts and plotted using the ggplot2 R-package (https://ggplot2.tidyverse.org).

### Immune responses to LPS in vivo.

Twelve-week-old female *Mvk*^VI/Δ91^ and control *Mvk^+/VI^* mice were injected i.p. with 100 μg LPS endotoxin (*E. coli* O111:B4, Sigma-Aldrich) and culled 2 hours later for collection of serum and peritoneal fluid (by lavaging the peritoneal cavity with 1 mL PBS). In separate studies, *Mvk*^VI/Δ91^ mice were pretreated with 50 mg/kg i.p. MCC950 (or saline) 1 hour prior to i.p. LPS administration, and serum collected 2 hours later. Baseline serum samples were prepared by bleeding mice 1 week before the experiment. Serum and peritoneal fluid were analyzed for levels of cytokines and chemokines using a multiplex immunoassay (Bio-Plex, Bio-Rad) and a MAGPIX platform (Luminex), according to the manufacturers’ instructions. IL-18 was measured using a mouse IL-18 ELISA (Abcam).

### Elevation of T_core_.

Mice were bled by retro-orbital venepuncture to obtain a pretreatment sample of plasma, and T_core_ was measured 1 week later using a rectal probe (the procedure taking less than 10 seconds per animal). Mice were given a 1 mL subcutaneous saline injection before heating for 18 hours at 38°C in a purpose-made chamber (2 mice per cage). In some instances, mice were allowed to recover in normal housing conditions at 22°C for 48 hours. Immediately after heat exposure, T_core_ was recorded and blood and spleens collected for analysis of plasma MA levels and uRab proteins, respectively.

### Statistics.

Details of the statistical analyses performed are stated in the figure legends. Data are presented as the mean ± standard deviation (SD) and *P* values were calculated using unpaired, 2-tailed Student’s *t* test with Welch’s correction, or 1-way ANOVA with Tukey’s post hoc test for multiple comparisons in GraphPad Prism v9. Significance was defined as a *P* value of less than 0.05: *P < 0.05, **P < 0.01, ***P < 0.001, ****P < 0.0001. Alternatively, where stated in the figure legend, data shown are representative of at least 3 independent experiments.

### Study approval.

Studies on patient blood samples was approved by the Sydney Children’s Hospitals Network Human Research Ethics Committee (HREC/18/SCHN/403). All subjects gave written informed consent in accordance with the Declaration of Helsinki. All experiments involving mice were approved by the Garvan Institute/St. Vincent’s Hospital Animal Ethics Committee (protocols 16/23 and 19/20).

## Author contributions

MAM, MJR, and OPS designed the experiments. MAM, OPS, JJ, YX, EKF, and KAP performed experiments and analyzed data. EMF performed flow cytometric analysis. EK and MPH performed mass spectrometry analysis. DGZ and RB performed CRISPR/Cas9 gene editing. EKD and AABR assisted with experimental design. SIOD and SK performed the protein structure analysis in Aquaria. SM, PH, CMMM, and AS acquired patient samples. MJR conceived ideas and oversaw the research program. MJR and MAM wrote the manuscript.

## Supplementary Material

Supplemental data

## Version 1 10/03/2022

Electronic publication
