# Supplementary material for: Increased core body temperature exacerbates defective protein prenylation in mouse models of mevalonate kinase deficiency
Source: J Clin Invest. 2022 Oct 3;132(19):e160929. doi: 10.1172/JCI160929 (PMC9525117; doi:10.1172/JCI160929)
Supplement: Supplemental data [file jci-132-160929-s052.pdf]

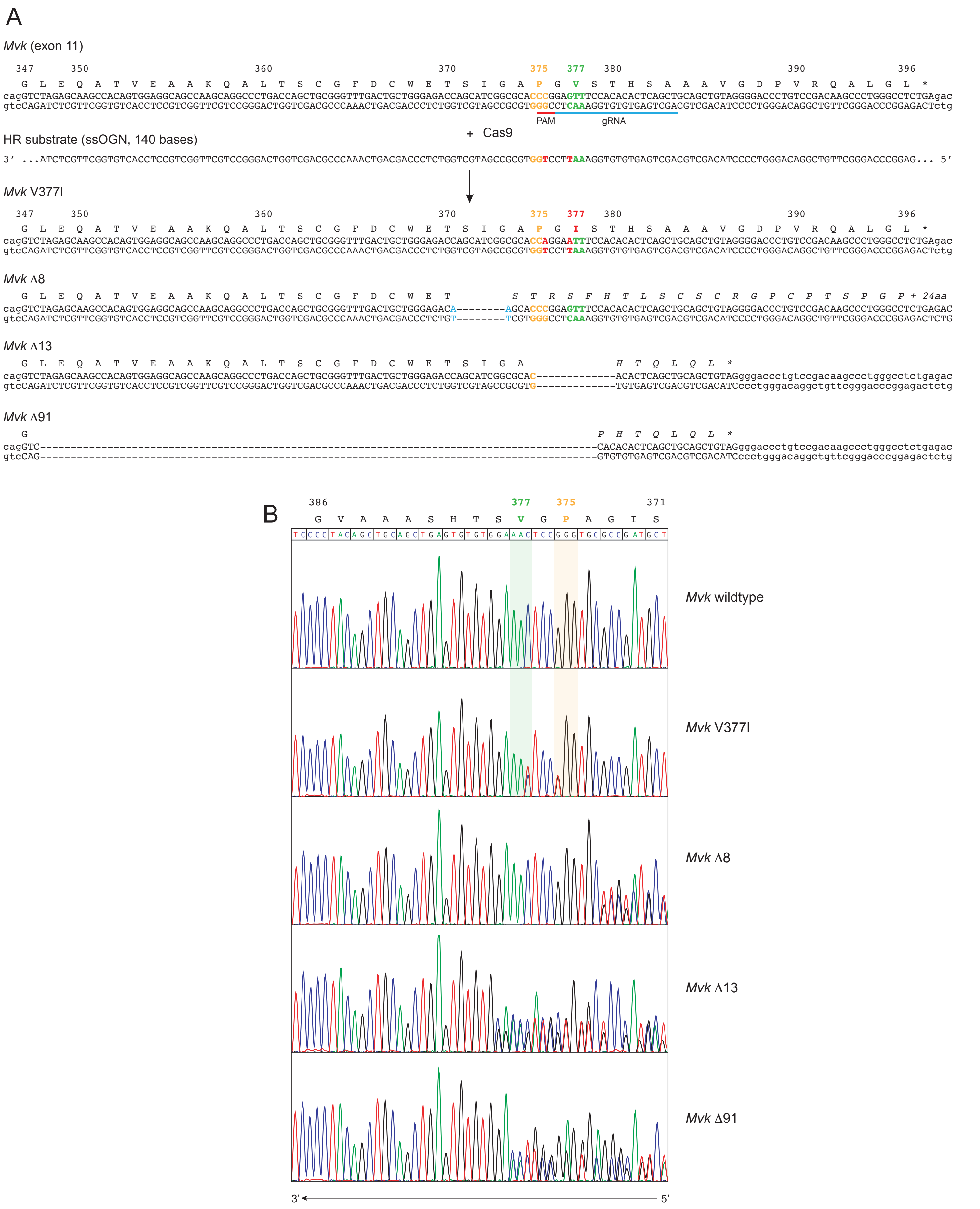

Supplemental Figure 1. A) DNA sequence of exon 11 of wild-type and mutant mouse *Mvk* genes. The encoded *Mvk* amino acid sequence is indicated above the central base of the corresponding codons in the wild-type allele.The 20 base sequence corresponding to the Cas9-bound guide RNA (gRNA) is shown together with the associated protospacer-associated motif (PAM) and the central portion of the 140 base, single stranded oligionucleotide (ssOGN) homologous recombination (HR) substrate indicated below the wild-type allele. Targeted P375 and V377 codons are shown in ochre and green respectively, with altered bases in the V377I mutant highlighted in red. Inserted bases in the Δ8 mutant allele are indicated in blue. B) Sanger sequencing of wild-type *Mvk* exon 11 and mice heterozygous mice for the V377I, Δ8, Δ13 and Δ91 mutant alleles.

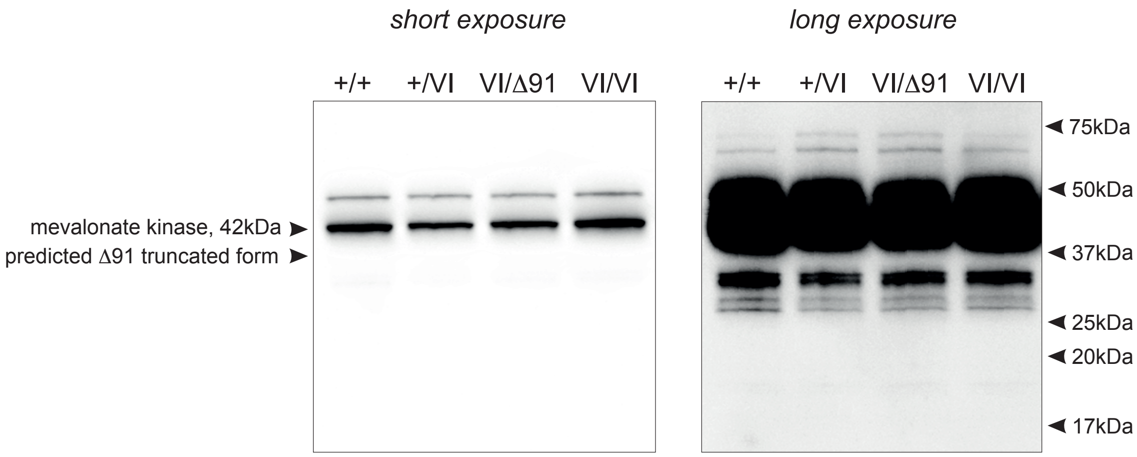

Supplemental Figure 2. Western blot of mevalonate kinase in liver homogenate from wildtype and Mvk mutant mice. Enhanced chemiluminescence detection with short exposure (left) and longer exposure (right) shows the full length 40 kDa isoform of mevalonate kinase in wildtype (+/+) liver and *Mvk* mutant liver. The Mvk^{VI} mutation does not detectably alter the mass of the major 42 kDa isoform of mevalonate kinase in Mvk^{VI} or $Mvk^{VI/VI}$ liver. A truncated form, predicted to be generated from the $Mvk^{VI/\Delta 91}$ mutation, or any degradation products could not be detected in $Mvk^{VI/\Delta 91}$ liver.

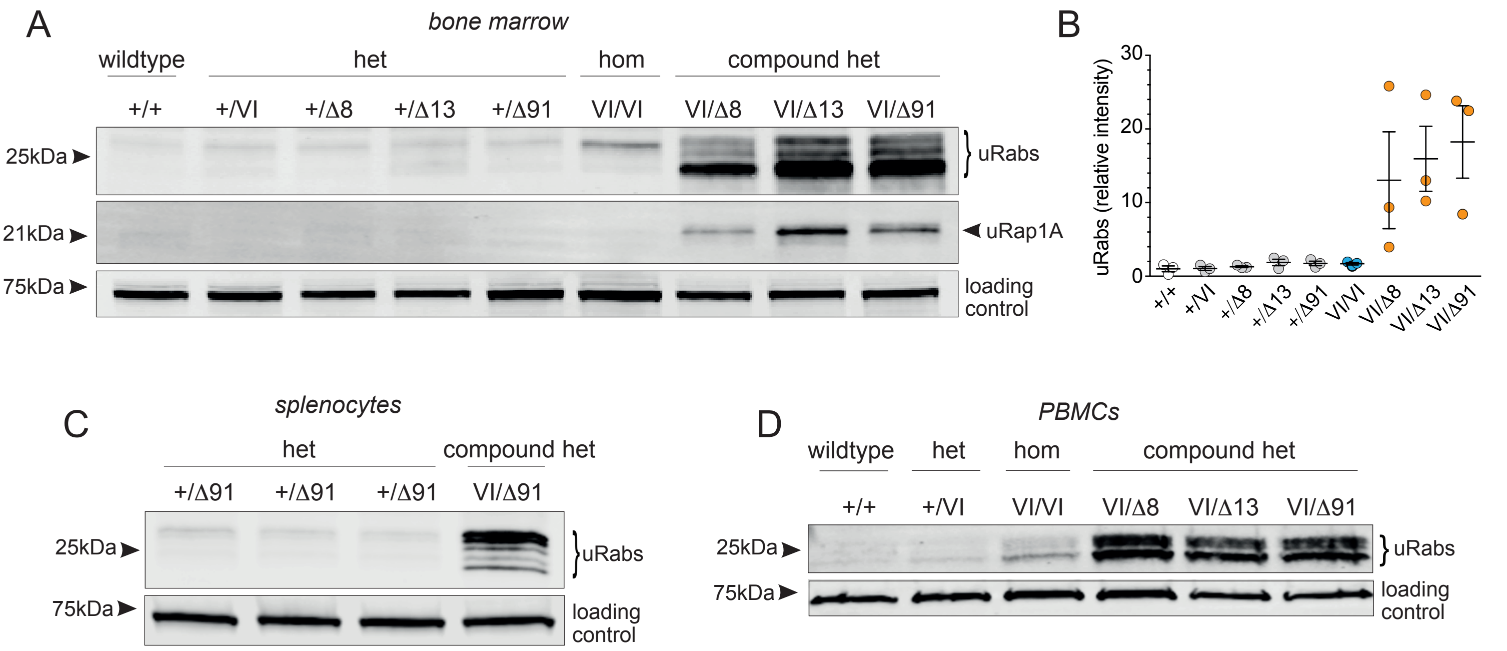

Supplemental Figure 3. The mevalonate pathway is defective in bone marrow cells, spleen cells, PBMCs and peritoneal cells from homozygous and compound heterozygous *Mvk* mutant mice. A) Detection of unprenylated Rab GTPases (uRabs) and unprenylated Rap1A (uRap1A) in bone marrow from *Mvk* mutant mice: homozygous $Mvk^{VI/VI}$ and three lines of compound heterozygous *Mvk* mutants compared to wildtype or heterozygous $Mvk^{+/VI}$ mice. B) Blots from prenylation assays of whole bone marrow samples were analysed by densitometry and values of uRabs normalised to the loading control (mean +/-SD, n=3 mice per genotype). Each symbol represents a single wildtype (white), heterozygous (grey), homozygous $Mvk^{VI/VI}$ (blue), or compound heterozygous (orange) mouse. Detection and comparison of uRabs in C) spleen cells from $Mvk^{VI/\Delta 91}$ and $Mvk^{+/\Delta 91}$ mice, and D) in PBMCs from *Mvk* mutant mice (homozygous $Mvk^{VI/VI}$ and three lines of compound heterozygous mice), wildtype and heterozygous $Mvk^{+/VI}$ mice. In panels A,C,D an endogenous, biotinylated 73 kDa protein was used as a loading control.

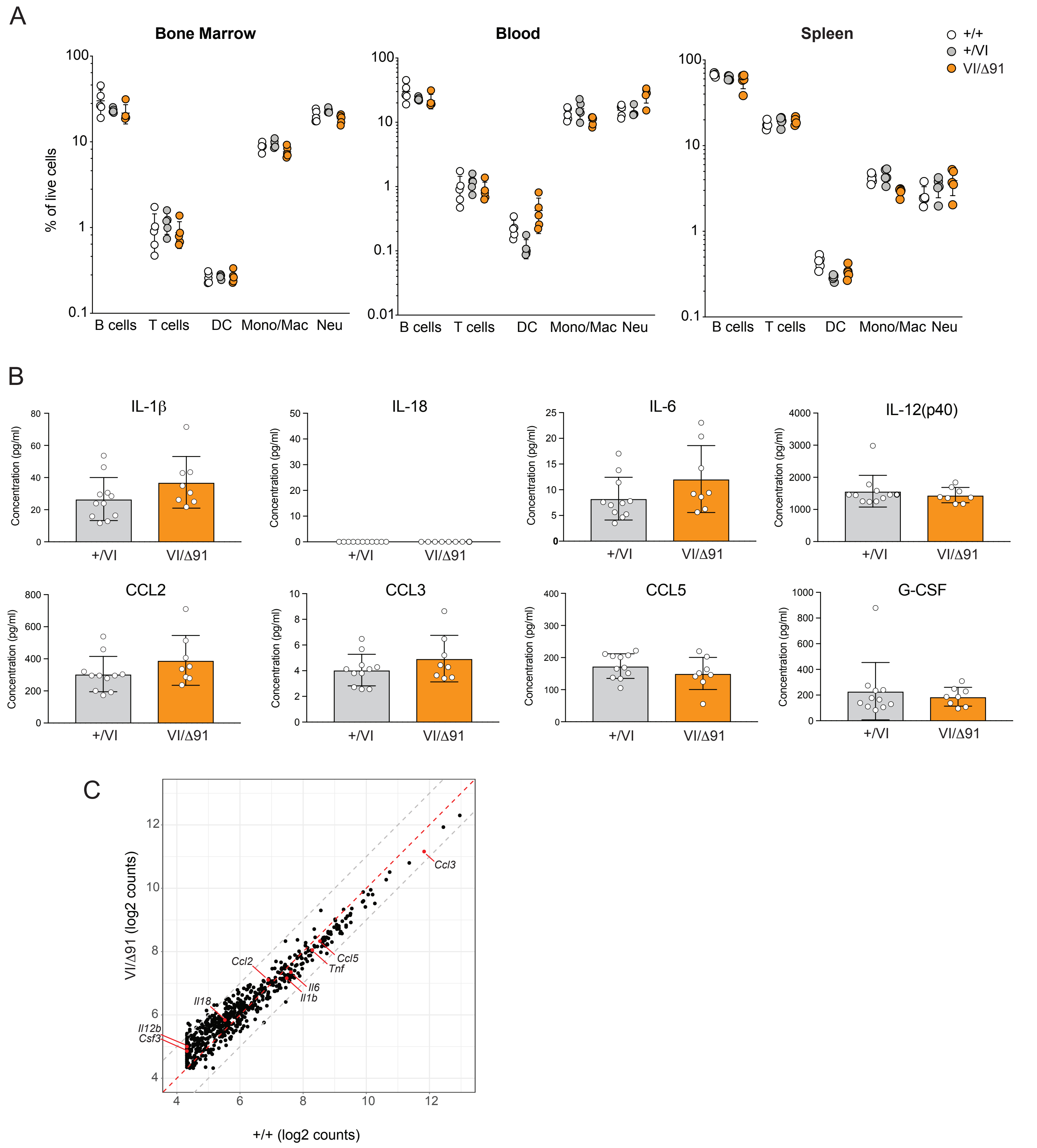

Supplemental Figure 4. Baseline immune cell populations, gene expression in PBMCs and serum cytokine concentrations in *Mvk*$^{+/VI}$ and *Mvk*$^{VI/\Delta 91}$ mice. A) B220^{+} B cells, TCRβ^{+} T cells, CD11c^{+} dendritic cells (DC), B220^{-} TCRβ^{-} CD11c^{-} CD11b^{+} Ly6G^{-} Ly6C^{+} monocytes/macrophages (Mono/Mac) and B220^{-} TCRβ^{-} CD11c^{-} CD11b^{+} Ly6G^{+} Ly6C^{int} neutrophils (Neu), expressed as percentage of live leukocytes (log scale) in the bone marrow (left), blood (middle) or spleen (right) of *Mvk*$^{+/+}$ (no fill), *Mvk*$^{+/VI}$ (grey fill) or *Mvk*$^{VI/\Delta 91}$ (orange fill) mice (n=5 per group). Values are mean +/- SD, differences were not statistically significant using a t-test corrected for multiple comparisons using the Holm-Sidak method. B) Cytokine and chemokine levels in serum samples from 12-week old female mice (n=11 *Mvk*$^{+/VI}$, n=8 *Mvk*$^{VI/\Delta 91}$). Cytokines were measured using a multiplex assay or a single cytokine ELISA for IL-18 (IL-18 levels were undetectable). Bars show mean +/- SD (not significantly different by ANOVA with Tukey's post-hoc test); each symbol represents a single mouse. C) Gene expression analysis in freshly isolated PBMCs from *Mvk*$^{+/+}$ and *Mvk*$^{VI/\Delta 91}$ mice using an nCounter Myeloid Innate Immunity 754-gene panel (Nanostring). Cytokine/chemokine genes measured in (B) by multiplex immunoassay are highlighted. Red dotted line indicates no change in gene expression between *Mvk*$^{VI/\Delta 91}$ (y axis) and *Mvk*$^{+/+}$ (x axis), and grey dotted lines above and below indicate a log2 fold change of 1 or -1, respectively.

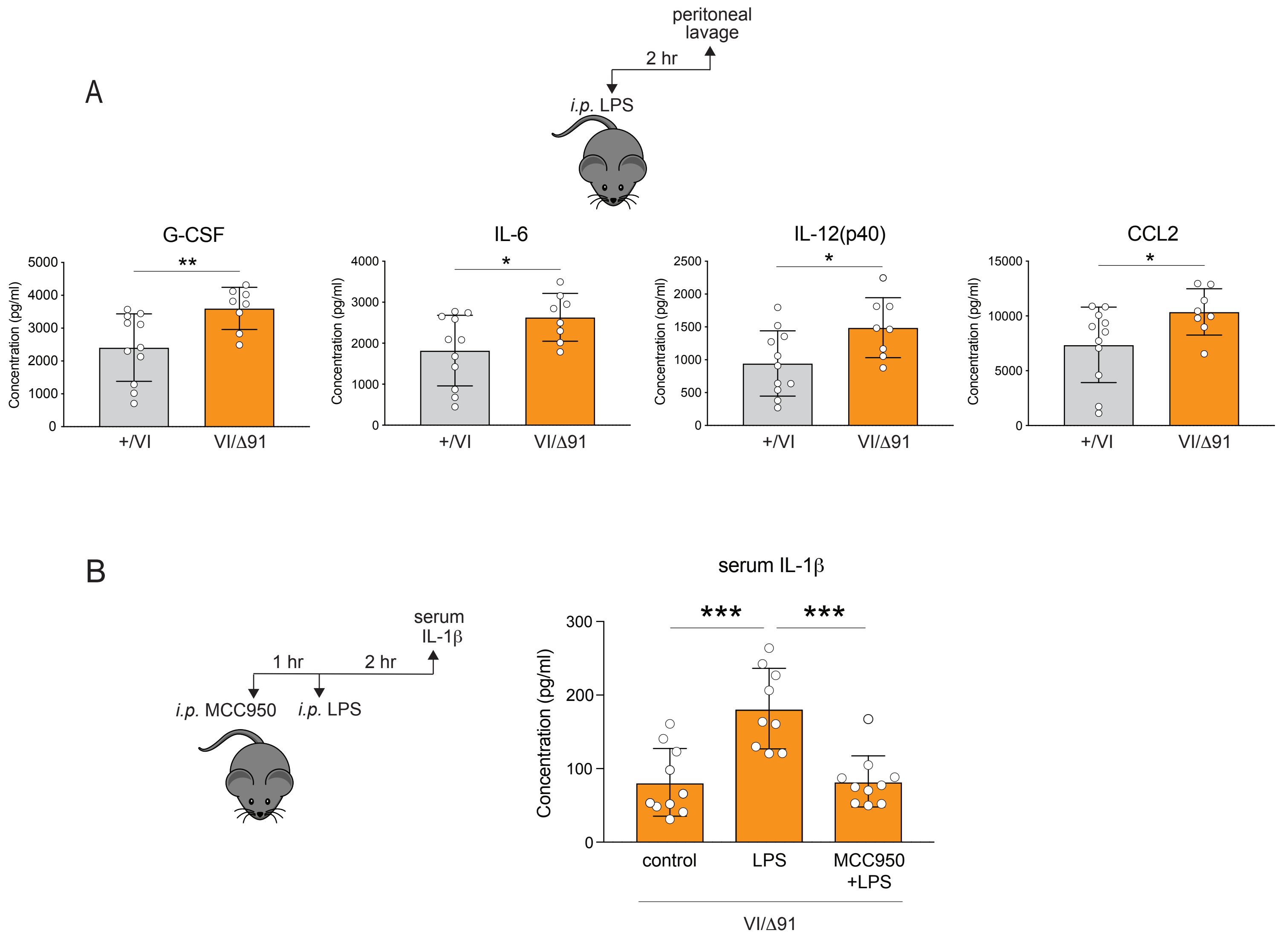

Supplemental Figure 5. Elevation of inflammatory cytokines and chemokines in peritoneal fluid and serum of $Mvk^{VI/\Delta 91}$ mice following *in vivo* LPS treatment. A) Peritoneal lavage fluid was collected from 12-week old female mice, 2 hours after *i.p.* injection of LPS (n=11 $Mvk^{+/VI}$, n=8 $Mvk^{VI/\Delta 91}$). Samples were analysed using a multiplex immunoassay. Bars show mean +/-SD (each symbol represents a single mouse), *p<0.05, **p<0.01, unpaired t-test with Welch's correction. B) Levels of IL-1β in serum samples from untreated (control) $Mvk^{VI/\Delta 91}$ mice (n=10), and $Mvk^{VI/\Delta 91}$ mice treated with *i.p.* LPS (n=9), or with 50mg/kg *i.p.* MCC950 prior to LPS treatment (n=10). Bars show mean +/- SD, ***p<0.001 (ANOVA with Tukey's post-hoc test); each symbol represents a single mouse.

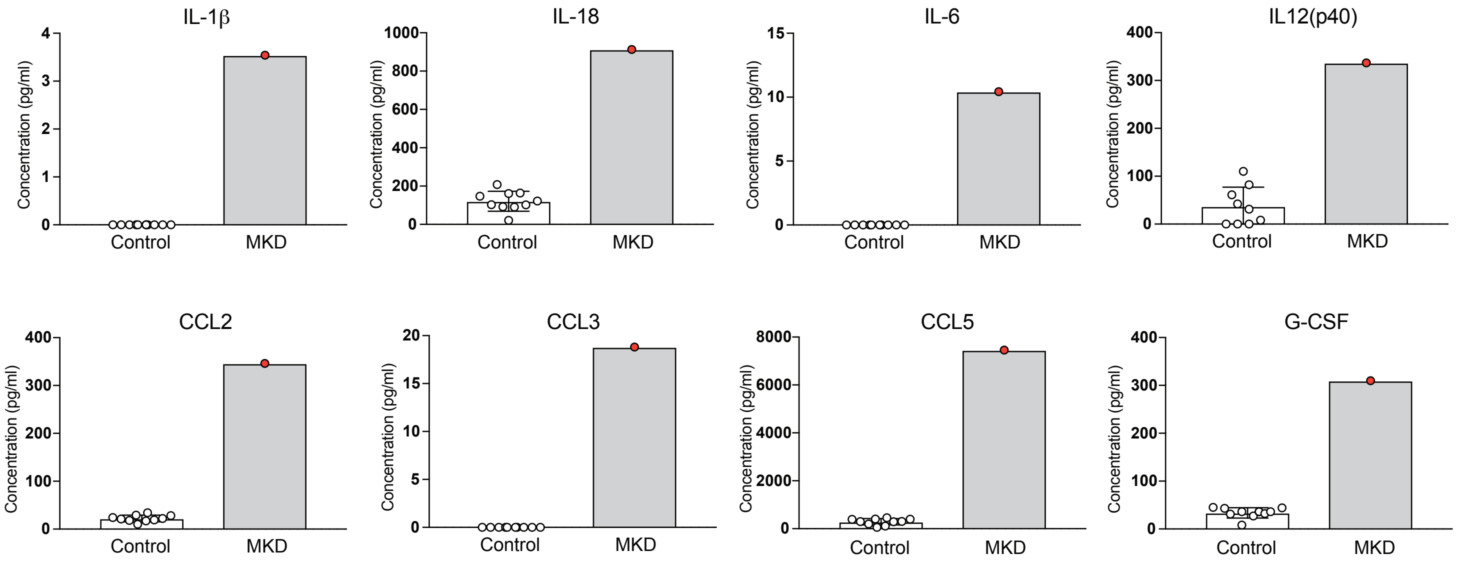

Supplemental Figure 6. Elevation of inflammatory serum cytokines and chemokines from a MKD patient compared to 10 healthy controls. The MKD individual is a compound heterozygous for the pathogenic *MVK* variants p.V377I/p.Tyr149_Ser150insAlaTyr, previously described in Munoz *et al*, *Front Immunol* 10:1900 (2019). Cytokines and chemokines were measured using a multiplex immunoassay or a single cytokine ELISA for IL-18 (each symbol represents a single individual).

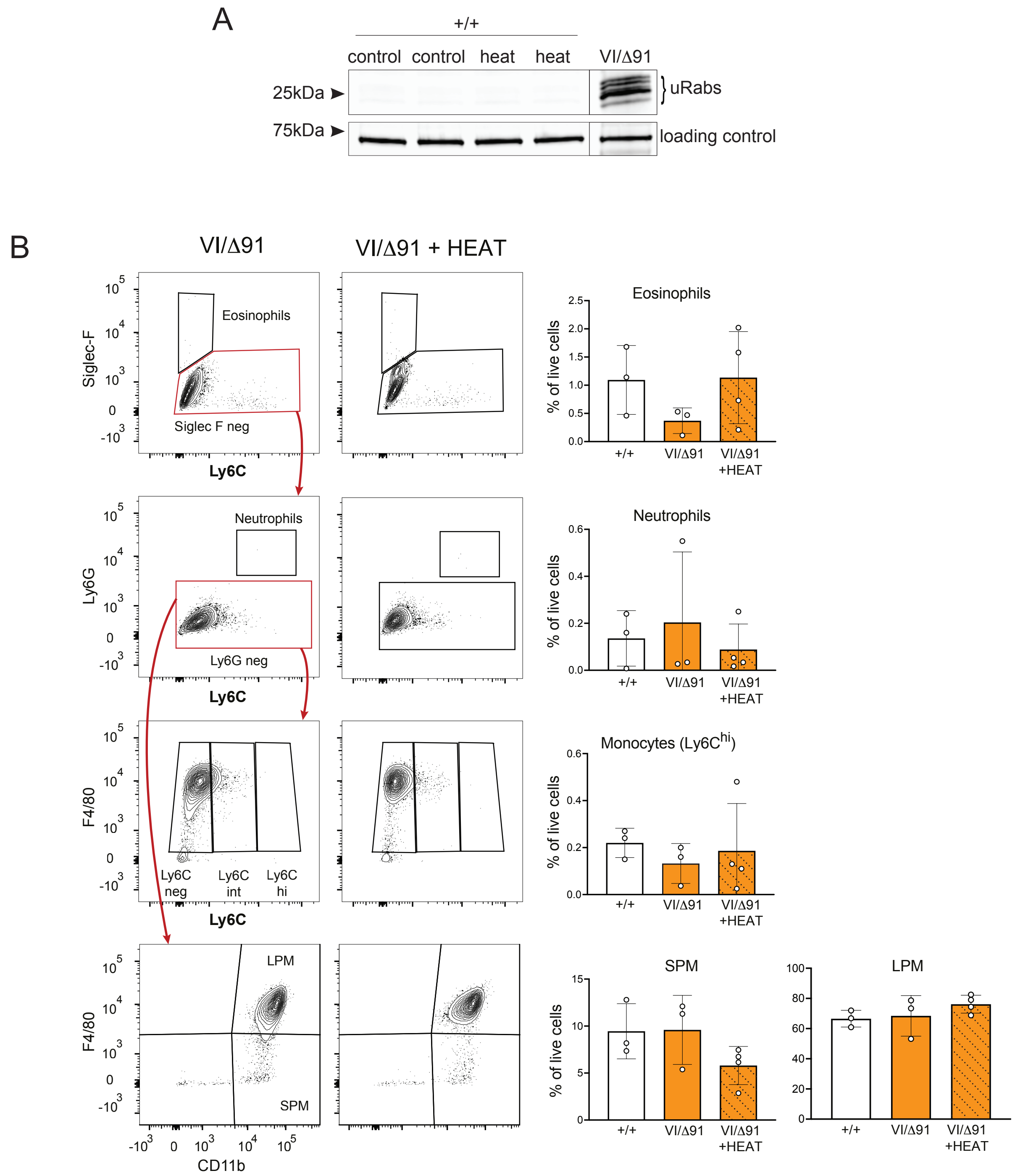

Supplemental Figure 7. Elevated ambient temperature does not affect protein prenylation in wildtype animals or cause acute inflammation in *Mvk*$^{VI/\Delta 91}$mice. A) Analysis of unprenylated Rab GTPases (uRabs) in spleen cells from two wildtype (+/+) mice maintained at 22°C or two mice housed at 38°C for 18 hours (heated). An endogenous, biotinylated 73 kDa protein was used as the loading control. Spleen cells from a *Mvk*$^{VI/\Delta 91}$mouse were used as a positive control on the same blot.
B) Representative FACS plots from peritoneal cells isolated from untreated wildtype (*Mvk*$^{+/+}$) and unheated and heated *Mvk*$^{VI/\Delta 91}$mice. Polygons in red depict the populations displayed in the proceeding plot (red arrow). Histograms (right) show relative abundance (percentage of live cell singlets) from wildtype (*Mvk*$^{+/+}$), unheated and heated *Mvk*$^{VI/\Delta 91}$ mice. Immune cell populations were identified as follows: eosinophils (TCRb^{-}, B220^{-}, CD11c^{-}, Siglec-F+); neutrophils (TCRb^{-}, B220^{-}, Siglec-F^{-}, Ly6G^{hi}); inflammatory monocytes (TCRb^{-}, B220^{-}, Siglec-F^{-}, Ly6G^{-}, Ly6C^{hi}), LPM (TCRb^{-}, B220^{-}, Siglec-F^{-}, Ly6G^{-}, F4/80^{hi}, CD11b^{hi}) and SPM (TCRb^{-}, B220^{-}, Siglec-F^{-}, Ly6G^{-}, F4/80^{+}, CD11b^{+}). Bars show mean +/- SD, each symbol represents a single mouse (n=3 for wildtype and *Mvk*$^{VI/\Delta 91}$ mice, n=4 for heated *Mvk*$^{VI/\Delta 91}$ mice).

Supplemental Table 1. Antibodies and reagents used for flow cytometry.

Label	Target	Clone	Dilution	Supplier	Cat #
BUV395	CD11b	M1/70	1/200	BD Biosciences	563553
BV421	F4/80	T45-2342	1/100	BD Biosciences	565411
BB515	Siglec-F	E50-2440	1/200	BD Biosciences	E50-2440
PE	CD11c	N418	1/200	eBiosciences	117307
APC	Ly6G	1A8	1/200	BD Biosciences	560599
BUV737	B220	RA3-6B2	1/300	BD Biosciences	612838
PE-Cy7	Ly6C	AL-21	1/200	BD Biosciences	560593
APC-Cy7	TCRB	H57-597	1/300	BD Biosciences	560656
FITC	CD11b	M1/70	1/200	BD Biosciences	17-0051-81
PerCP/Cy5.5	CD11c	N418	1/200	Thermo Fisher	45-0114-82
PE/Cy7	Ly-6G	1A8	1/200	BD Biosciences	560601
Biotin	Ly-6C	AL-21	1/200	BD Biosciences	557359
BV510	CD19	6D5	1/100	BioLegend	115546
BV786	CD86	GL1	1/100	BD Biosciences	740877
BV605	CD44	IM7	1/300	BD Biosciences	563058
PerCP/Cy5.5	CD62L	MEL-14	1/200	BD Biosciences	560513
Fc block	CD16/CD32	2.4G2	1/200	BD Biosciences	553142
7AAD	Dead cells	-	1/500	Invitrogen	A1310
Zombie Aqua	Dead Cells	-	1/500	Biolegend	423101
